# Surface Oxygen Depletion of Layered Transition Metal Oxides in Li-Ion Batteries Studied by *Operando* Ambient Pressure X-ray Photoelectron Spectroscopy

**DOI:** 10.1021/acsami.2c19008

**Published:** 2023-01-09

**Authors:** Anna T.S. Freiberg, Simon Qian, Johannes Wandt, Hubert A. Gasteiger, Ethan J. Crumlin

**Affiliations:** † Chair of Technical Electrochemistry, Department of Chemistry and Catalysis Research Center, Technical University of Munich, Garching bei MünchenD-85748, Germany; ‡ Advanced Light Source, Lawrence Berkeley National Laboratory, Berkeley, California94720, United States; § Chemical Sciences Division, Lawrence Berkeley National Laboratory, Berkeley, California94720, United States

**Keywords:** *operando* X-ray photoelectron spectroscopy, lithium-ion battery, layered transition metal oxide, oxygen depletion, phase transition

## Abstract

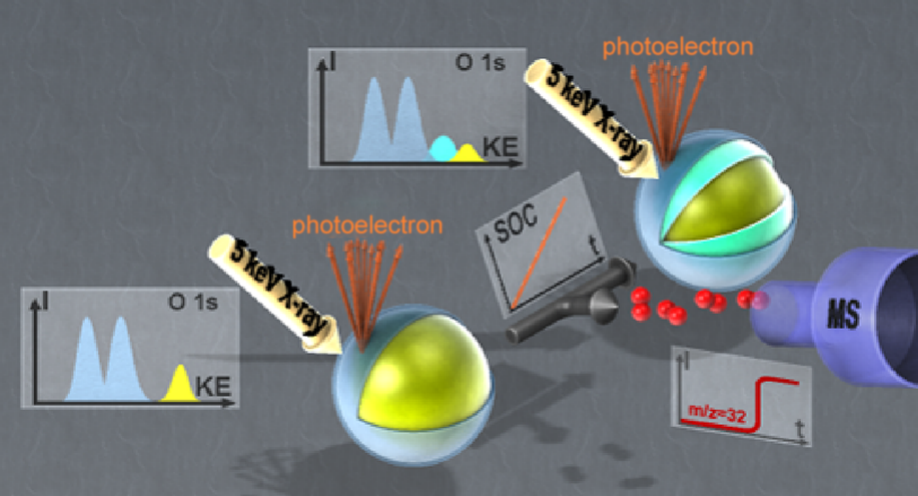

A new *operando* spectro-electrochemical setup was developed to study oxygen depletion from the surface of layered transition metal oxide particles at high degrees of delithiation. An NCM111 working electrode was paired with a chemically delithiated LiFePO_4_ counter electrode in a fuel cell-inspired membrane electrode assembly (MEA). A propylene carbonate-soaked Li-ion conducting ionomer served as an electrolyte, providing both good electrochemical performance and direct probing of the NCM111 particles during cycling by ambient pressure X-ray photoelectron spectroscopy. The irreversible emergence of an oxygen-depleted phase in the O 1s spectra of the layered oxide particles was observed upon the first delithiation to high state-of-charge, which is in excellent agreement with oxygen release analysis via mass spectrometry analysis of such MEAs. By comparing the metal oxide-based O 1s spectral features to the Ni 2p_3/2_ intensity, we can calculate the transition metal-to-oxygen ratio of the metal oxide close to the particle surface, which shows good agreement with the formation of a spinel-like stoichiometry as an oxygen-depleted phase. This new setup enables a deeper understanding of interfacial changes of layered oxide-based cathode active materials for Li-ion batteries upon cycling.

## Introduction

Li-ion batteries are the key technology in mobile devices as laptops and cell phones, as well as being the primary energy storage technology used in battery electric vehicles for decarbonization of the private transportation sector.^1^ As a working electrode material, mixed transitional metal (*M*)-layered oxides (i.e., Li_1+*x*_[Ni_*a*_Co_*b*_Mn_*c*_]_1–*x*_O_2_)^2−5^ are used almost exclusively due to their high power and energy density and satisfactory cycle life.^6,7^ The latter is achieved, however, by limiting the upper cut-off voltage—and therewith the amount of extractable lithium—as complete delithiation leads to (partial) restructuring and collapse of the layered oxide material, along with oxygen evolution from the crystal lattice in the near-surface region. The concurrent polarization resistance increase in layered oxides that have undergone high degrees of delithiation severely limits their power density and power efficiency and, consequently, cycle-life.^4,5,8−10^

While oxygen evolution was observed during the first few cycles to high cut-off voltages for all layered oxide materials that exceed roughly 80% state-of-charge (SOC),^8,11−13^ studying the formed oxygen-depleted surface phase is difficult and can be observed only after prolonged cycling. The techniques that are ordinarily used like X-ray diffraction,^14−16^ transmission electron microscopy, and electron diffraction^15,17−19^ can resolve oxygen-depleted surface phases only if they are sufficiently thick and/or if the formed phase is crystalline. Due to its high surface sensitivity, X-ray photoelectron spectroscopy (XPS), however, is able to detect single-digit percentage level quantities of oxygen-depleted phases on the surface of layered oxide particles and does not require a well-ordered crystalline structure that diffraction-based techniques rely on and that probably form in Li-ion battery electrodes only over time, long after the oxygen has already evolved from the particle surface.

XAS in TEY mode has been used to study the transition metal oxidation state at the surface of cycled layered-oxide-based cathode active materials *ex-situ*, which could be directly correlated to oxygen-release and changes in the O K-edge characteristics.^20,21^ A quantification of the surface phase stoichiometry, however, is not possible by such means due to the strong overlay of multiple oxygen-containing species including surface impurities and electrolyte decomposition products. Information on the relative intensity of different oxygen-containing surface near components is available by XPS and can therewith provide additional insights into compositional surface changes of layered oxide cathode materials upon cycling.

In an earlier study, we reported the occurrence of an additional metal oxide peak in the O 1s spectrum of a layered transition metal oxide at binding energies higher than the *M*O_2_ peak after prolonged cycling to higher cut-off voltages.^14^ Based on measurements of different transition metal oxide references, we assigned this additional peak to the formation of an oxygen-depleted phase similar to a spinel or rock-salt structure, as done by Lebens-Higgins et al.^22^ To study the oxygen depletion occurring during the first delithiation of layered transition metal oxide materials, a new *operando* setup had to be designed. This setup allows the collection of SOC-resolved data, while avoiding artifacts by sample transfer (including possible time-dependent processes) and preparation of highly sensitive delithiated electrodes.

*Operando* XPS has been used to study electrochemical interfaces employing various setups and configurations.^23^ For standard high vacuum systems, solid-state setups (sometimes in conjunction with ionic liquids)^24−27^ and closed cells with a thin analysis window that also acts as a current collector for the studied electrode material have been used.^28−30^ The major drawback of such setups is the transfer of those finding to the understanding of the active material/electrolyte interface in standard Li-ion battery cells, which seriously limits the ability to characterize cathode active materials.

To study electrochemical interfaces of electrodes with gases and liquids, analysis chambers with differential pumping have been developed, which allow the pressure at the experimental section of the chamber to be kept at reasonably high levels.^31−36^ This technique is commonly referred to as ambient pressure XPS (APXPS), and it is especially advantageous when studying fundamental aspects of the electrochemical reactions of gases on electrodes (i.e., lithium-air batteries^37,38^) or the interfacial chemistry of model electrodes in different solvents with the so-called dip-and-pull method.^35,39−44^ While the latter is very well suited to examining fundamental questions about interfacial chemistries, control of the thickness of the liquid film meniscus on top of the pulled-out electrode is challenging (and for active materials that have micro- to nanoparticle shapes thought to be impossible) and it is limited to low current density regimes.

Another important development is the transition of the probing X-ray beam from the soft X-ray (<2 keV photon energy) to the tender X-ray regime (≈2–6 keV). This change significantly increases the probing depth and allows the gathering of information from solid/liquid interfaces, buried interfaces, and the near-surface “bulk”.^23,35^ In our study, the estimated probing depth for the O 1s orbital—based on the inelastic mean free path of the photoelectrons—into the active material is roughly 7 nm for an incident X-ray beam energy of 5 keV; this is more than double the probing depth obtained by a soft X-ray source, such as Al Kα (see the Supporting Information, section S3 for further depth and thickness discussion).

To study the oxygen depletion of NCM111 upon delithiation, we designed a new *operando* electrode setup that provides nominal electrochemical performance, a controlled electrolyte film thickness on the probed NCM111 particles, and the capacity to operate at reduced pressures while maintaining a stable liquid film to enable adequate probing depth into the active material.

## Experimental Section

### Battery MEA Design

Accessing of the X-ray beam and detecting emitted photoelectrons, while also ensuring a good electrical and ionic connection of the probed NCM111 particles, required the construction of a fuel cell-like membrane electrode assembly (MEA) design.

NCM111 (Li_1+*x*_[Ni_1/3_Co_1/3_Mn_1/3_]_1–*x*_O_2_, BASF, Germany) was chosen as a working electrode material. Prior to ink preparation, the NCM111 powder was dried at 300 °C for 30 h in a vacuum glass oven (Büchi, Switzerland). The dried powder was put into an 8 mL HDPE bottle filled with roughly 12 g of 3 mm_dia_ ZrO_2_ beads and an equivalent of Super C65 conductive carbon (Timcal, Switzerland) in a mixture of 90/6 by weight and mixed on a roller mill at 100 rpm for 1 h. Commercial LITHion dispersion (11%_wt_ in iso-propanole (IPA)) was used as the ion-conducting polymer (being a cation-exchange ionomer) and added to the NCM/C65 mixture, resulting in a total solid ratio of NCM/C65/LITHion of 90/6/4. The solid content was adjusted to  by addition of pure IPA (>99.8% “anhydrous”, Sigma Aldrich, <100 ppm H_2_O based on Karl-Fischer Titration). The ink was further mixed on a roller mill at 60 rpm for 5 h. The ink was coated onto 50 μm virgin PTFE foil (Angst+Pfister, Germany) at a wet-film thickness of 80 μm using the Mayer rod technique, resulting in an average loading of the electrodes of . The coating was dried at 50 °C for at least 5 h.

As a counter electrode, delithiated LiFePO_4_ (LFP 400, BASF, Germany) was used. The powder was chemically delithiated to a nominal stoichiometry of Li_0.1_FePO_4_ using aqueous K_2_S_2_O_8_ solution as described in an earlier study.^45^ In short, the LFP powder is given into a round-bottom flask with 0.07M K_2_S_2_O_8_ solution under constant argon flow at room temperature, whereas the delithiation stoichiometry of the LFP is set by the ratio of the added powder and the volume of the solution. The suspension was stirred for 20 h. Afterward, the powder was filtered off and washed with ultrapure water (>18 MOhm cm, MilliQ, Merck, Germany). Drying of the powder was done in an oven at 70 °C overnight followed by drying in a vacuum glass oven equipped with a cooling trap at 200 °C for at least 5 h. The ink for the LFP counter electrode had a weight ratio of LFP/C65/LITHion of 80/4/16 and was mixed in a planetary mixer (THINKY, USA) at 2000 rpm. To increase the viscosity of the ink, the LITHion dispersion was first thickened by partial evaporation of the IPA to a solid content of 15%. The powders were given into the mixing cup with half of the ionomer solution and mixed for 5 min. After addition of the second half of the needed ionomer solution, the ink was mixed again for 5 min. The ink was spread onto a carbon cloth (CC-G8 w/o PTFE, Quintech, Germany) at a wet-film thickness of 500 μm resulting in an average loading of . The LFP counter electrodes were dried at 50 °C for at least 5 h and compressed with 100 MPa before hot-pressing.

Li-ion-exchanged Nafion HP (20.3 μm, reinforced) was used as the membrane. Details on the ion-exchange of the membrane can be found in an earlier publication.^46^ In short, after boiling the membrane in 1 M HNO_3_ and washing it with ultrapure water, ion-exchange is performed by immersing the membrane in saturated, boiling LiOH solution for 8 h, followed again by washing with ultrapure water and drying of the membrane at room temperature overnight.

Battery MEAs were fabricated using the hot-press approach. Circular NCM electrodes with a diameter of 15 mm were paired with 17 mm diameter LFP electrodes. The ion-exchanged membrane was punched to 28 mm_dia_. The stack made up of the LFP counter electrode, two layers of the ion-exchanged membrane, and the NCM working electrode was placed between two sheets of Kapton foil, and two pieces of PacoPads (type #5500, Pacothane Technologies, USA) were used as a pressure distribution medium. Hot-pressing of the MEA was executed at 205 °C under a pressure of 1.5 MPa for 10 min. The PTFE decal was removed from the NCM electrode afterward and weighed to get the precise NCM loading of each MEA. The pressed MEAs were placed between two glass plates and dried at 130 °C in a vacuum glass oven for 12 h before transfer to the glovebox for cell assembly.

### APXPS Cell Assembly

**Figure 1:**
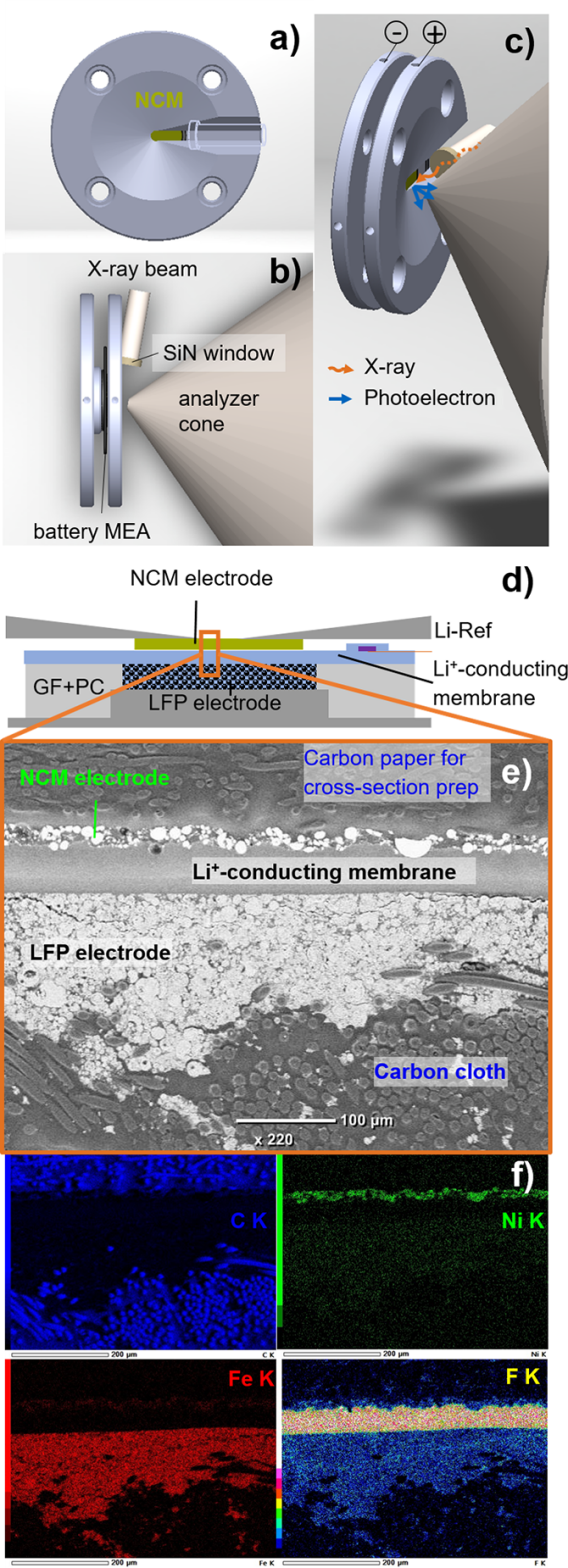
*Operando* APXPS and cell setup. The cell holder, including compression springs and screws, is omitted for clarity. (a) Front view of electrochemical cell with conical slit to accommodate the photoelectron beam outlet close to the NCM electrode. (b) Top view of the spectroelectrochemical cell, including the battery MEA with beam outlet and analyzer cone position. (c) 3D arrangement of the spectro-electrochemical cell, the X-ray beam, and the analyzer cone. (d) Cross-sectional sketch across the spectroelectrochemical cell showing the position of the lithium reference electrode and the PC reservoir in the GF separator. (e) SEM image of the polished cross section of the battery MEA embedded in epoxy resin. (f) EDX maps of carbon, nickel, iron and fluorine of the SEM image shown in panel e.

The MEAs were assembled into the APXPS *operando* cell in an argon-filled glovebox (MBraun, <10 ppm H_2_O, <30 ppm O_2_). A schematic of the cell appears in Figure 1. The MEA is soaked with propylene carbonate (PC, >99.9% “anhydrous”, Sigma, <100 ppm H_2_O based on Karl-Fischer Titration), which is given into a reservoir volume filled with glass fiber separator rings (GF, dried for 3 days at 300 °C in a vacuum glass oven; 250 μm microfiber filter 691, VWR). The rings, with an inner diameter of 20 mm and an outer diameter of 28 mm, were centered by a circular step with a height of 2 mm at the bottom plate of the XPS *operando* cell, on which the LFP side of the MEA was placed. Eight GF rings were used, which were filled with 1 mL of PC solvent, and another 20 μL of PC was given on top of the NCM electrode. A lithium-metal reference electrode was added by placing a small piece of lithium foil (roughly 2 mm_dia_, 450 μm, 99.9%, Rockwood Lithium, USA) at the edge of the membrane and a piece of copper foil leading outside of the cell. The cells were left for soaking for 12 h to ensure good ionic conductivity. After soaking, the cell was either cycled inside of the glovebox or given into a small plastic bag for transfer to the beamline, for which the time between removal from the glovebox and attaching the cell to the analysis chamber under counterflow of argon was kept to a minimum (<1 h). Cycling of the cells was done using a Biologic potentiostat (Biologic Science Instruments, France) at room temperature.

### OEMS Cell Assembly and Measurement

To study the gas evolution when cycling the NCM111 battery MEA, we used our on-line electrochemical mass spectrometry (OEMS) setup, which is explained in detail in earlier studies.^13,47^ The Li-reference electrode is not used in that setup, and the NCM111 potential is controlled vs the delithiated LFP electrode. Please refer to section S7 of the Supporting Information for additional experimental details.

Two different cycling procedures were studied in the OEMS system, one a continuous constant current cycling at C/30 (referenced to a nominal NCM111 capacity of 275 mAh/g, with a rate of 1C corresponding to 275 mA/g) to evaluate the overall performance of the battery MEA as well as an intermittent cycling procedure at C/10 in 20% SOC intervals, each followed by an 1 h OCV hold to mimic the electrochemical procedure during the *operando* APXPS measurement. To ensure complete delithiation, the cells were cycled to an upper cut-off potential of the NCM111 working electrode of 5.2 V vs Li^+^/Li. Relithiation was executed to a lower cut-off potential of 1.3 V vs Li^+^/Li.

### *Operando* APXPS Analysis

The *operando* APXPS measurements were performed at Beamline 9.3.1 (bending magnet beamline) of the Advanced Light Source at the Lawrence Berkeley National Laboratory (Berkeley, USA). The X-ray energy was set to 5 keV, and the spot size was roughly 0.3 mm in diameter, which is small compared to the XPS-accessible area of the NCM111 electrode (2 mm x 8 mm). Our goal is to study the oxygen depletion from the layered oxide material, which happens at the buried interface below the electrolyte film. Therefore, the kinetic energy of the photoelectrons must be high enough to make it through the polymer film to the analysis cone, meaning that the tender X-ray regime (≈2–6 keV) is optimal for this study.

Beamline 9.3.1 is equipped with a HiPP-2 differential pumping stage. After insertion of the *operando* APXPS cell into the analysis chamber under argon counterflow, the system was pumped down without additional backfilling of inert gas. The APXPS cell was tightly wrapped laterally with cleanroom wipes (VWR), which were soaked with PC to limit the amount of solvent removed from the cell through the open slit between both current collector plates, and secured by Parafilm. An additional PC reservoir was put into a small beaker placed just below the cell in the analysis chamber. After the system was pumped down, the pressure within the analysis chamber was 30–40 Pa, i.e., still above the vapor pressure of PC (<8 Pa at room temperature^48,49^).

To obtain XPS spectra at different SOCs and thus to follow the evolution of an oxygen-depleted surface phase of the NCM111 material in the first cycle, we used the electrochemical procedure that consisted of cycling at C/10 in 20% SOC intervals, followed by an OCV period (roughly 1.5 h each) during which the spectra were recorded. After 15 min in OCV, the O 1s, Ni 2p_3/2_, Co 2p_3/2_, Mn 2p_3/2_, C 1 s, and a second O 1s spectra were recorded, corresponding to an irradiation time of roughly 70 min. The first O 1s spectrum obtained was used for further analysis, as it had not been impaired by beam-induced damage of the polymer (see the Supporting Information, sections S5 and S6 for discussion). At 0% SOC, 100% SOC, and at the end of discharge, the S 1s and F 1s spectra were also recorded. Beam-induced damage of the polymer was observed, which in extreme cases led to a loss in sensitivity toward the peaks associated to the active material (see section S6 in the Supporting Information for details). Therefore, the shutter was kept closed during the electrochemical procedure and opened only just before the XPS measurement. In addition to this, the cell was moved relative to the beam for each set of spectra, so as to change the irradiated spot of the NCM material within the conical slit, thus ensuring that the spectra were always recorded on electrode locations that had not experienced any beam damage.

The NCM111 working electrode was grounded to the analyzer to establish energy levels and to eliminate any uncertainty due to the applied electrochemical potential. All spectra discussed within the main text of this study are aligned to the C 1s of the C65-conductive carbon additive (BE_aligned_ = 284.8 eV) in the working electrode, resulting in an energy correction of 0.16 eV ± 0.1 eV, which is largely to account for the photon energy of the beamline. Although spectra were recorded during the OCV period of the electrochemical cycling procedure, the potential was still relaxing slightly during spectra collection, which led to small energy shifts of the electrically insulating electrolyte components. A discussion of this can be found in the Supporting Information (section S5). A Shirley background was used throughout data analysis.

## Results and Discussion

### *Operando* Setup

The setup for the *operando* APXPS measurements and the detailed structure of the battery MEA are shown in Figure 1. The cell hardware consists mainly of two stainless steel circular plates acting as current collector plates for the working and counter electrodes. The top plate, which is in direct contact to the NCM electrode, has a milled conical slit (2 mm width) to allow proximity to the incoming X-ray beam as well as to the XPS analyzer cone (Figure 1 a–c). The cell compression of ≈2200 hPa is achieved by springs on the back of the cell (omitted in the figure for clarity). The analysis chamber of the beamline is continuously pumped down, whereas the atmosphere is filled with PC vapor from both a reservoir placed just below the electrochemical cell and from PC-soaked wipes wrapped laterally around the cell (not shown).

A cross section of the battery cell is shown as a scheme in Figure 1d, and a scanning electron microscopy (SEM) image and EDX (energy-dispersive X-ray spectroscopy) maps of the cross section of an MEA are provided in Figure 1e,f. For details on the cross section preparation and measurements, please refer to section S7 in the Supporting Information. The cross section shows that the maximum thickness of the NCM electrode (≈5-8 μm) consists of two primary particles, as can also be estimated from the loading. This low loading reduces (interparticular) inhomogeneity throughout the NCM electrode with respect to SOC, as the ionic resistance for the delithiation of all particles is similar; at the same time, a percolating electrical pathway exists that should allow full usage of the NCM capacity even within the slit of the current-collector plate (see later discussion). The LFP electrode on the other hand has a film thickness of roughly 150 μm, and additional electrode material can be found within the pores of the carbon cloth substrate. This substrate was chosen to minimize the electrical resistance within the counter electrode while ensuring a sufficiently high lithiation capacity. Having a closer look at the EDX maps shown in Figure 1f reveals that the distribution of the component specific elements (nickel, iron and fluorine) is as predicted, and that the fluorine representing the polymer electrolyte is well distributed throughout both electrodes (Figure 1).

### Physical Properties of the Battery MEA

The Supporting Information (sections S1–S3) provides a detailed discussion on the choice of solvent and the ionic conductivity of the battery MEA, as well as an estimate of the polymer electrolyte film thickness and corresponding probing depth. The estimated film thickness of the PC saturated polymer (<11 nm) that is covering the active material particles and the inelastic mean free path of photoelectrons from the O 1s orbital (7–9.5 nm) are very close, meaning that the XPS data obtained truly represent the outermost layer of the cathode active material particles (see the Supporting Information, section S3 for further discussion).

To directly probe the NCM particles by XPS, parts of the current collector plate of the working electrode have to be cut out (see Figure 1a). Consequently, the probed NCM particles need to have good electrical contact by a percolating carbon network in the *in-plane* direction of the working electrode. Simple polarization measurements in dependency of the ratio between the directly contacted part of the geometric electrode area and the electrode area connected electrically only *in-plane* can be found in the Supporting Information, section S2. These measurements show that, even when 50% of the electrode are not covered by the current collector plate (i.e., for an 11 mm diameter hole in the upper current collector plate), it has no significant impact on the overpotential and achievable capacity of the NCM electrode up to a C-rate of up to C/10; i.e., there is no major interparticular electrical resistance *in-plane*. In the final *operando* setup, less than 9% of the geometrical electrode area remains open for spectroscopy (i.e., uncovered by the upper current collector plate), which suggests that the NCM particles probed by XPS are in an electrochemical state representative of the whole electrode in the *in-plane* direction. Still, single particles might have poorer electrical connections to the carbon network, and this could be independent of whether they are in the open area of the NCM electrode or just below the current collector plate but close to the membrane interface. As a result, inhomogeneity in SOC might be observed spectroscopically and do occur at higher C-rates based on the oxygen release analysis via OEMS, even though the overall electrochemical performance is not impaired (see section S2 of the Supporting Information for detailed discussion).

### OEMS Gassing Analysis of the NCM Battery MEA

The goal of the *operando* APXPS study is to observe directly the evolution of an oxygen-depleted phase at the surface of the NCM particles, which requires an examination of the oxygen evolution characteristics of the NCM111 material in the MEA setup.

The NCM MEA with the Li_0.1_FePO_4_ counter electrode is placed into our OEMS cell, including the PC reservoir stored in the GF rings below the membrane. The lithium reference electrode is not used in this case, and the potential of the NCM WE is calculated assuming an LFP overpotential as measured in the symmetrical MEA setup shown in section S1 of the Supporting Information. Please refer to section S7 of the Supporting Information for a detailed discussion of the OEMS results.

Two different electrochemical procedures are used: one involves a continuous constant-current charge and discharge at C/30, and the other consists of intermittent cycling at C/10 in steps of 20% SOC with an 1 h rest after each step to mimic the electrochemical procedure used in the *operando* APXPS experiment.

**Figure 2:**
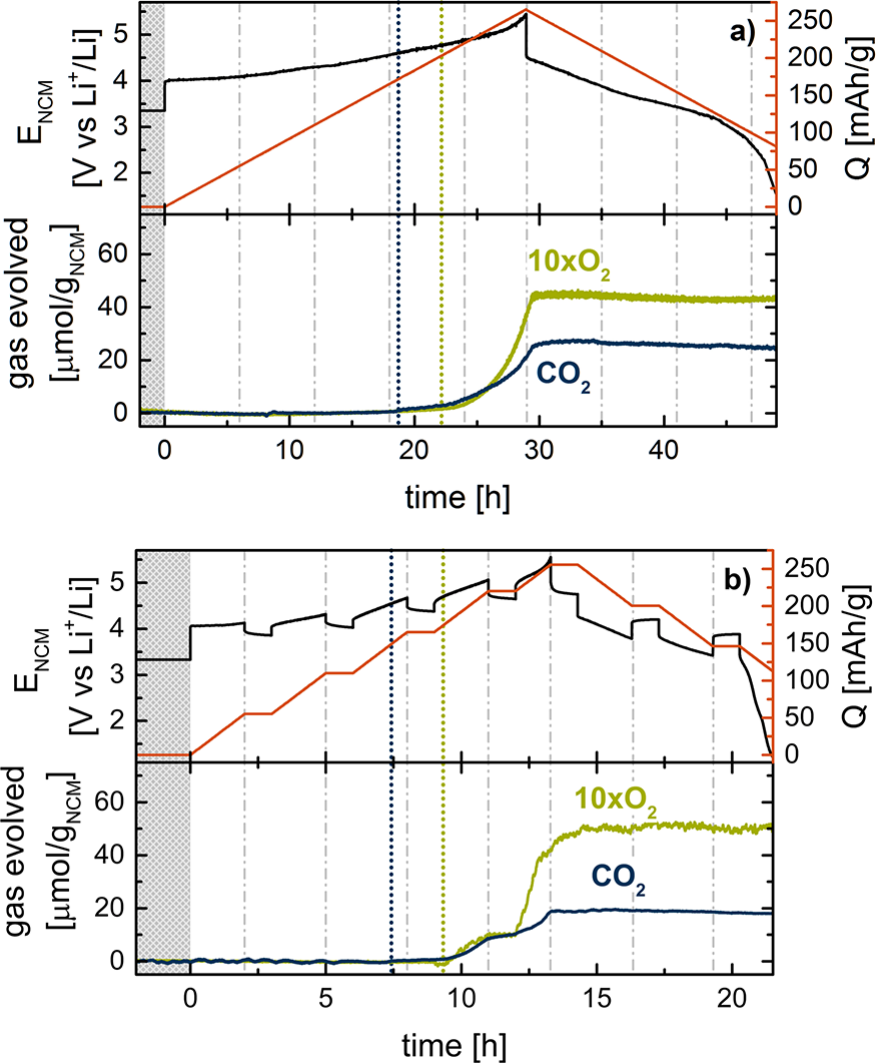
Gas evolution analysis of NCM111 in the *operando* MEA configuration cycled vs Li_0.1_FePO_4_ at 25 °C under argon in the OEMS cell. Top panels: potential profile (black, left scale) and specific capacity (orange, right scale). Bottom panels: cumulated evolved gas amount of O_2_ (green) and CO_2_ (blue). (a) Continuous charge and discharge at C/30. (b) Intermittent charging at C/10 in 20% SOC intervals. Vertical dotted lines correspond to the onset of O_2_ (green) and CO_2_ (blue) evolution.

The top panels of Figure 2 show the electrochemical response of the MEA for those two different cycling procedures, with the potential shown as a black line (left axis) and the specific capacity shown as an orange line (right axis). During charge, the MEA that is cycled continuously at C/30 (Figure 2a) delivers roughly 262 mAh/g_NCM_ during charge and 195 mAh/g_NCM_ during discharge. With 255 mAh/g_NCM_ charging capacity, the MEA cycled at C/10 with intermittent OCV periods (Figure 2b) has an only slightly lower delithiation capacity but a significantly lower relithiation capacity (136 mAh/g_NCM_). Because, in general, relithiation of the NCM particles is possible in the battery MEA setup at small C-rates, we ascribe this low reversibility to an increased charge transfer resistance for relithiation of NCM in the PFSA-based electrolyte. The electrochemical characteristics are discussed further in the next section.

The OEMS gas evolution characteristics of the MEA cycled at C/30 in Figure 2a are as expected with an O_2_ onset at roughly 75% SOC.^8,12,13^ When cycling the MEA at C/10 in Figure 2b, CO_2_ is evolved in the common potential range^13,50−52^ (predominantly stemming from anodic oxidation of the organic carbonate solvent), whereas the oxygen evolution is already observed at the beginning of the charging step from 60% SOC to 80% SOC. This earlier gassing onset shows that, in the battery MEA setup, a C-rate of C/10 already leads to local inhomogeneity of the SOC of the NCM111 electrode.

Based on the OEMS gas evolution analysis of the battery MEA, we expect the *operando* APXPS measurement to show an earlier evolution of the oxygen-depleted phase compared to an electrode cycled more slowly or in a setup with better ionic and/or electrical conductivity. Because the overall volume of evolved gas during intermittent cycling at C/10 is similar to that from standard cells and similar to that at the lower C-rate of C/30, we anticipate no differences in the nature of the information gathered by XPS. The electrochemical response of the battery MEA with respect to the cycling procedure is studied further in the following section.

### Polarization of the NCM Battery MEA

**Figure 3:**
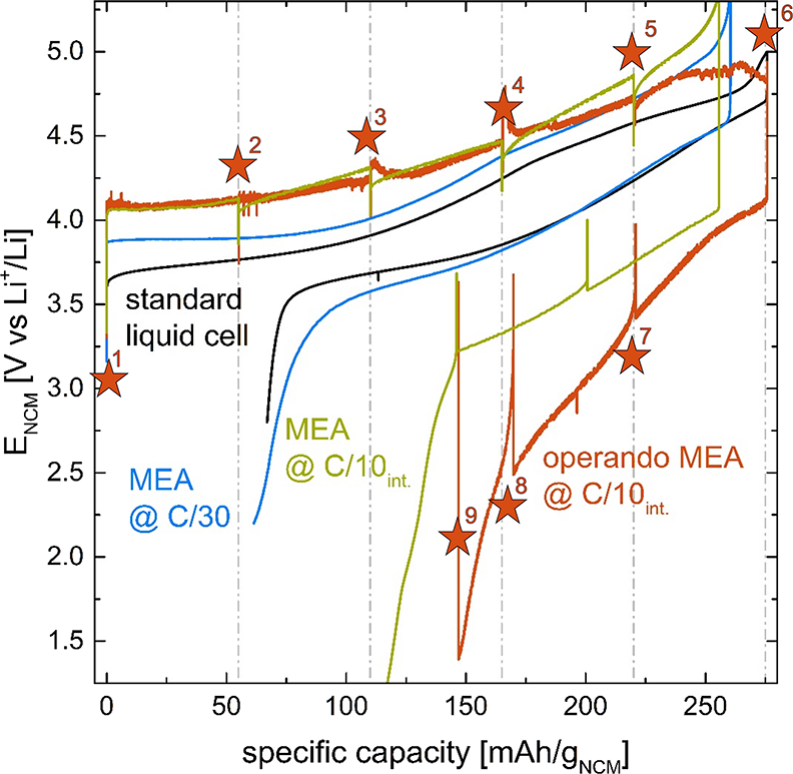
Voltage vs capacity characteristics of the NCM111 MEA under different cycling conditions compared to a standard liquid electrolyte cell (black line) measured vs the lithium reference electrode. The battery MEA at C/30 (blue line) and at C/10 in 20% SOC intervals (green line) acquired at ambient pressure (under argon), and the data of the *operando* APXPS cell at C/10 in 20% SOC intervals during the XPS measurements (orange line) are compared. Orange stars along with their numbers indicate points at which XPS spectra were obtained.

As shown in the electrochemical response of the NCM battery MEA in the OEMS setup, we observe no major deviations from standard cells at low C-rates. At higher C-rates, however, the conductivity of this MEA setup seems to lead to inhomogeneity with respect to the local SOC, and an increased resistance during relithiation is observed, leading to only roughly 2/3 of the discharge capacity at C/30. For the *operando* APXPS measurement, the reduced pressure required might also lead to higher ionic resistances because parts of the PC absorbed in the membrane might be removed faster than the amount of solvent that can diffuse into the polymer from the reservoir. We therefore compare the electrochemical response of a standard liquid electrolyte cell using a standard electrode configuration and 1.5 M LiPF_6_ in PC as an electrolyte (for experimental details, see the Supporting Information, section S7) with that of the battery MEA in the APXPS cell setup, cycled either at ambient pressure at C/30 or using the intermittent cycling protocol at C/10 either at ambient pressure or during the actual *operando* measurement in the sample chamber at below ambient pressure. The electrochemical response of these cells is shown in Figure 3, along with the performance curve obtained during the *operando* APXPS measurement. The obtained capacities under these different conditions are given in Table 1. Except for the *operando* APXPS measurement, the average capacity along with the standard deviation of three-independent measurements is given.

**Table 1:** Comparison of the Specific Capacities of the NCM111 MEA at Different Cycling Conditions vs a Standard Liquid Electrolyte Cell (See the Supporting Information, Section S7)^a^

setup	*Q*_charge_[mAh/g_NCM_]	*Q*_discharge_[mAh/g_NCM_]
standard (C/30)	275 ± 2	205 ± 2
MEA (C/30)	263 ± 5	195 ± 4
MEA (C/10 int)	255 ± 5	135 ± 4
*operando* MEA	275	128

a The average of three-independent measurements is shown along with the standard deviation (except for the *operando* measurement).

As already observed in the OEMS setup, cycling of the battery MEA at C/30 leads to polarization characteristics very close to the liquid electrolyte cell reference, as shown in Table 1 and Figure 3 (blue line vs black line). Accelerating the cycling to C/10 with intermittent OCV periods (green line) leads to increased polarization during relithiation, which predominantly limits the discharge capacity. Very similar charging behavior can be seen for the *operando* MEA recorded during the XPS measurements (orange line in Figure 3), whereas the polarization at the end of charge flattens out. The charge is terminated based on a time limit corresponding to 275 mAh/g_NCM_. The discharge characteristics resemble the reference measurement at C/10 with intermittent OCV periods cycled in the glovebox but exhibits a higher overpotential and therefore slightly lower relithiation capacity. The higher polarization is due to the membrane drying out over time, as the PC continuously evaporates due to the reduced pressure in the APXPS analysis chamber or due to an overall lower PC content of the ionomer at the reduced pressure.

While the electrochemical performance during the *operando* APXPS measurement is limited predominantly during discharge, we expect the spectroscopic results to give meaningful information, especially during the charging of the NCM material. Due to the flattening of the potential profile toward the end of charge, it is not absolutely certain that 100% SOC was reached for the *operando* MEA. Based on the polarization similarities between the *operando* MEA and MEA cycled with the same C/10 intermittent cycling procedure at ambient pressure under argon and especially the similar discharge capacity (128 mAh/g vs 135 ± 4 mAh/g, respectively), we expect to have reached at least 90% SOC. The reason for the flattening of the potential profile at high SOC is not known at this time.

### *Operando* Ambient Pressure X-ray Photoelectron Spectra

**Figure 4:**
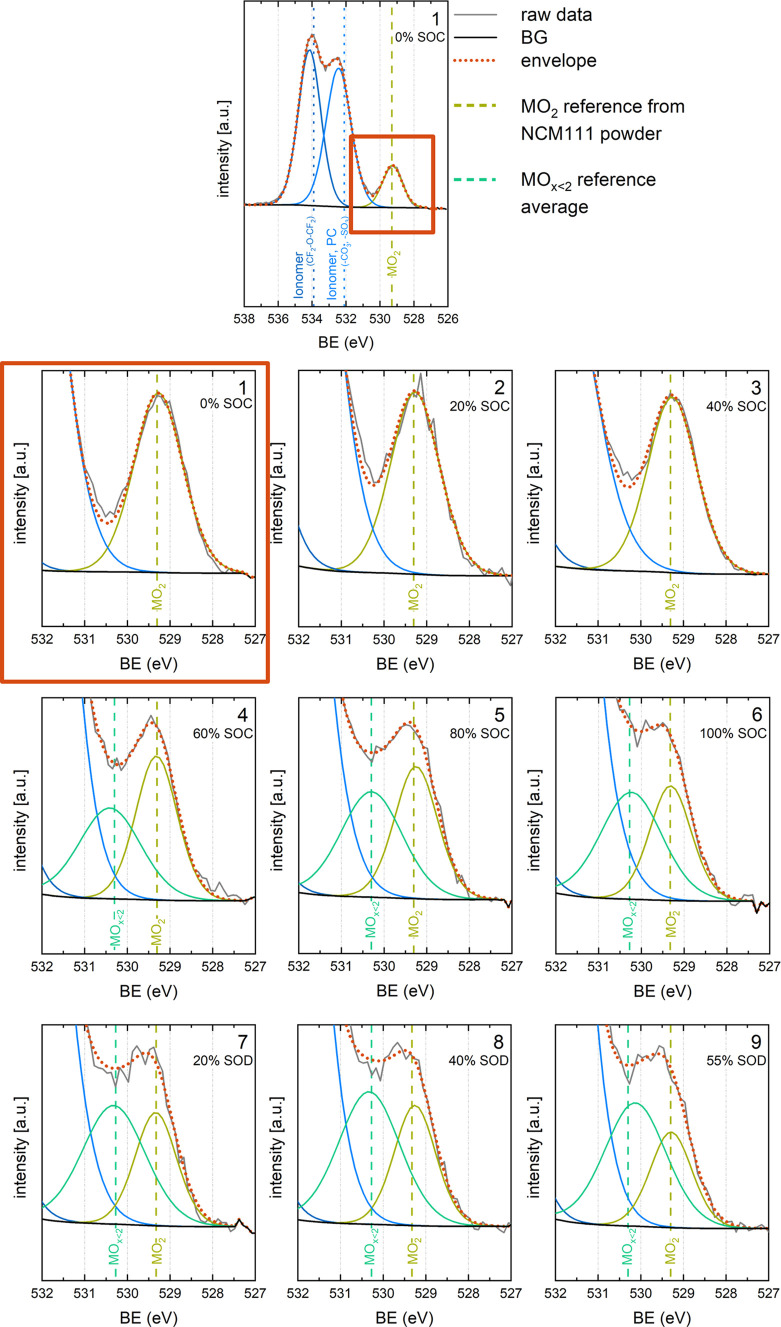
O 1s spectra of the *operando* NCM MEA at different SOCs, as indicated in Figure 3 that shows the associated voltage vs capacity curves (orange star symbols, numbered 1–9). *Ex-situ* references are indicated as dashed lines for the metal oxide and dotted lines for the electrolyte. All spectra are energy normalized by the C 1s of the conductive carbon.

The *operando* NCM111 MEA was charged and discharged in intervals of 20% SOC, followed by a rest of roughly 1.5 h to record spectra (the charge/discharge data are shown by the orange line in Figure 3). The acquired O 1s spectra are shown in Figure 4. While the complete O 1s spectrum of the pristine electrode (0% SOC) is shown, our emphasis is on the metal oxide region (orange rectangle), and therefore, only the zoom into the low binding energy region is shown for the other *operando* spectra. Additional information on the peak fitting and the reference data for the peak assignment can be found in the Supporting Information in sections S4 and S5. The full O 1s spectra of all steps during cycling are shown in the Supporting Information, section S5. From these, a constant probing depth into the active material can be inferred as discussed in the Supporting Information in section S3.

As shown in the top-most panel of Figure 4, the pristine NCM111 electrode in the battery MEA shows three distinct peaks. Based on references shown in the Supporting Information (section S4) and in the literature;^43,53^ we assign the peak at the highest binding energy (BE) to the −CF_2_–O–CF_2_– group of the polymer electrolyte, as well as to the C=O moiety of the carbonate group in the PC molecule (534.0–534.4 eV, dark blue line in Figure 4). The FWHM of this peak stays around 1.6–1.7 eV through all spectra, and it shifts slightly throughout cycling (further shown and discussed in the Supporting Information in section S5).

The peak centered around 532.2–532.6 eV (blue line in Figure 4) is assigned to the Li_2_CO_3_ surface impurity commonly found on NCM particles,^54^ as well as to the −SO_3_ group of the polymer^53^ and the C–O–C oxygen of the PC solvent.^43^ The peak positions of all references measured for those components are very close (see the Supporting Information, section S4), making a more detailed fitting of this region essentially impossible, as additional shifts due to charge corrections further complicate the analysis. Therefore, an FWHM of 1.7–2 eV, as obtained by using one single feature, is considered acceptable. Slight shifts in the position of this peak for different SOCs are also observed (see the Supporting Information, section S5).

Because both high energy peaks barely change in intensity and shape at different SOC intervals during the *operando* measurement, focus is set on the region in the O 1s spectra, where metal oxides can be observed and for which the spectra in Figure 4 are normalized to the overall intensity of the metal oxide features (i.e., to the area sum of the *M*O_2_ and *M*O_*x*<2_ peaks). The full O 1s spectra are provided in the Supporting Information, section S5. During charge, spectra were recorded between 0 and 100% SOC in 20% SOC intervals (spectra 1–6). During discharge, spectra were obtained that correspond to 20% state-of-discharge (SOD, 80% SOC, spectra 7), 40% SOD (60% SOC, spectra 8), and 55% SOD (45% SOC, spectra 9).

The metal oxide region is well separated from the features of the polymer, solvent, and the Li_2_CO_3_ impurity. The peak around 529.3 eV BE (green line) in the pristine electrode fits well with the reference for the layered metal oxide with the stoichiometric composition *M*O_2_ (dashed vertical green line). For the spectra obtained between 0% SOC and 40% SOC (panels 1–3 in Figure 4), only the *M*O_2_ peak has to be added to the background signal from the electrolyte to get a good fit of the acquired spectra. The *M*O_2_ peak does not move with polarization of the cell and keeps an FWHM of less than 1.4 eV. We do note a small change in the spectral shape around 530.5 eV, which is already present at 20% SOC and 40% SOC, but an additional peak representing the oxygen-depleted surface phase already at low SOCs cannot be reasonably added to the fit. This shape change is believed to be caused, rather, by the slight shifts in the polymer electrolyte features upon polarization (see the Supporting Information, section S5 for detailed discussion) and/or electrolyte decomposition products (see the Supporting Information, section S6).

Starting from the 4th spectrum corresponding to 60% SOC, an additional dominant shoulder forms between the *M*O_2_ peak and the low BE feature from the electrolyte. Fitting this part requires adding to the analysis an additional peak, which is centered roughly 0.8–1.1 eV higher in BE compared to the *M*O_2_ peak (turquoise line in Figure 4). Based on several references (see section S4 of the Supporting Information), this additional feature arising at slightly higher BE compared to the layered metal oxide peak can be assigned to an oxygen-depleted phase like a spinel (*M*_3_O_4_) or a rock-salt structure (*M*O). Because it is not possible to differentiate between those two structures based on XPS and because the structure formed is still largely debated in literature for different NCM materials, we will for now simply denote this phase as oxygen-depleted or *M*O_*x*<2_ (a structural discussion follows later). The average BE at which spinel and rock-salt structures are observed is indicated by a dashed vertical turquoise line. There is a random slight shift in the peak position for the *M*O_*x*<2_ phase, but it is always moving within a window of 0.2 eV and maintains an FWHM of 1.75 eV or less, which are reasonable values for a poorly defined and structured phase. A table summarizing the position and FWHM of all components in the O 1s spectra throughout the *operando* APXPS measurement appears in section S5 of the Supporting Information.

The additional feature that represents an oxygen-depleted phase arises after charging the NCM electrode to 60% SOC, and its intensity rises continuously throughout cycling in comparison to the layered oxide feature. During discharge of the MEA (panels 7–9 in Figure 4), the feature remains.

An additional O 1s feature emerging during the first charge of the battery to high potentials does not necessarily have to reflect the formation of an oxygen-depleted layer. Such additional features could also stem from electrolyte oxidation products of the cathode electrolyte interphase (CEI) that could still consist of oxide-containing compounds. In this battery MEA setup, the possible anodic oxidative decomposition products are limited as no conductive salt is used; however, decomposition products of the PFSA and PC could be present. As shown in the literature, decomposition products from carbonate solvents might be present in the O 1s spectra, as they are composed of C=O and C–O groups, but they would appear at a significantly higher BE of 531.5 eV and above,^22,55^ which means that they should not interfere with the quantification of the oxide (*M*O_2_ and *M*O_*x*<2_) region in the O 1s spectra. The anodic oxidative decomposition of PFSA in aprotic media is not studied well in literature.

To exclude any possible interference of electrolyte decomposition with the analysis of the oxide region in the *operando* O 1s spectra, we conducted a simplified model experiment in which we charged a pure C65 electrode in the same MEA configuration up to 5.2 V vs Li^+^/Li (see the Supporting Information, section S6). In this case, no additional feature in the low BE region of the O 1s signals could be observed, and only a minor broadening of the electrolyte-assigned region was noted, which could explain the change of the shape of the *operando* O 1s spectra of the NCM111 electrode at low SOC. The possible beam-induced damage of the electrolyte membrane over prolonged irradiation of several hours is shown in the Supporting Information (section S6), showing no interference with the oxide region in the O 1s spectra either, in accordance with the literature.

A comparison of the *operando* measurements and *ex-situ* XPS data collected at a standard high-vacuum system using Al Kα radiation is given and discussed in the Supporting Information, section S8, where comparable results with respect to oxygen-depleted phase formation have been obtained.

A better understanding of the relative evolution of the *M*O_*x*<2_ phase requires analysis of the area below the fits of the layered oxide and oxygen-depleted oxide phase. While the relative intensity of the metal oxides to the electrolyte-based peaks changes slightly (and can in general change due to beam damage, see sections S5 and S6 of the Supporting Information), the oxygen-depleted phase is formed out of the layered structure. Even though only the few outermost layers of the NCM111 particles are probed by the photoelectron analyzer (see sections S3 and S5 of the Supporting Information), the evolution of the relative share of the *M*O_*x*<2_ phase close to the surface of the particles remains meaningful because the film thickness of the electrolyte on the NCM111 particles is carefully controlled by the MEA design. An estimate of the probing depth variation for the different spectra throughout cycling of the NCM111 MEA possibly caused by a variation in polymer electrolyte film thickness due to a change in the PC content is given in section S3 of the Supporting Information. For all spectra containing the oxygen-depleted phase feature in the O 1s, the variation in electrolyte thickness is estimated to be less than 15%.

Removal of oxygen from the lattice will also change the ratio of transition metal to oxygen (*M*:O), which is why we give an estimate of this value by using the integrated area of the Ni 2p_3/2_ spectrum, along with relative sensitivity factors obtained from measurement of the bare NCM111 powder and the known structural formula of equal amounts of all transition metals, *n*_M_ = *n*_Ni_ + *n*_Co_ + *n*_Mn_ = 3*n*_Ni_. This value is then referenced to the area below the metal oxide peaks. A detailed explanation can be found in the Supporting Information, section S5. The nickel spectra are low in intensity, so that we have estimated the upper and lower limit of the true Ni 2p_3/2_ intensity based on the noise level (see section S4 of the Supporting Information) to estimate the possible range of the oxygen-depleted phase that might have formed.

**Figure 5:**
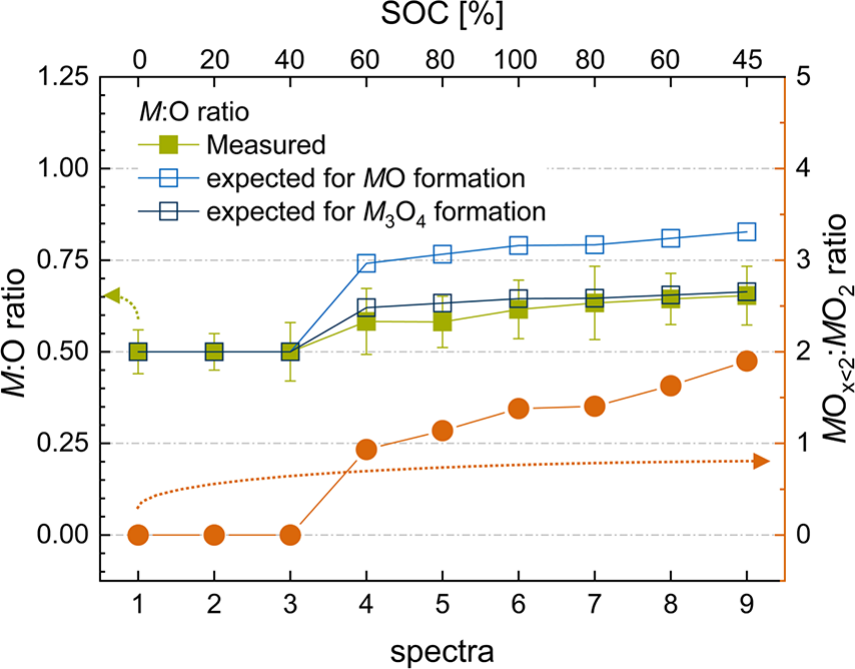
Area ratio of the O 1s XPS peaks of the oxygen-depleted *M*O_*x*<2_ phase and the pristine layered *M*O_2_ structure (orange circles, right axis) during the first cycle of the *operando* MEA. Ratio of *M* to oxygen at the respective points of the first cycle based on the O 1s features assigned to the metal oxides and the full area of the Ni 2p_3/2_ spectra (green, filled squares, left axis). Expected *M*:O ratio based on the *M*O_*x*<2_:*M*O_2_ ratio, assuming formation of a spinel phase (*M*_3_O_4_, dark blue hollow squares, left axis) or formation of a rock-salt phase (*M*O, blue hollow squares, left axis).

The evolution of the relative share of the *M*O_*x*<2_ phase (i.e., area ratio of *M*O_*x*<2_ to *M*O_2_) and the estimated *M*:O ratio of the probed NCM material are displayed in Figure 5.

**Figure 6:**
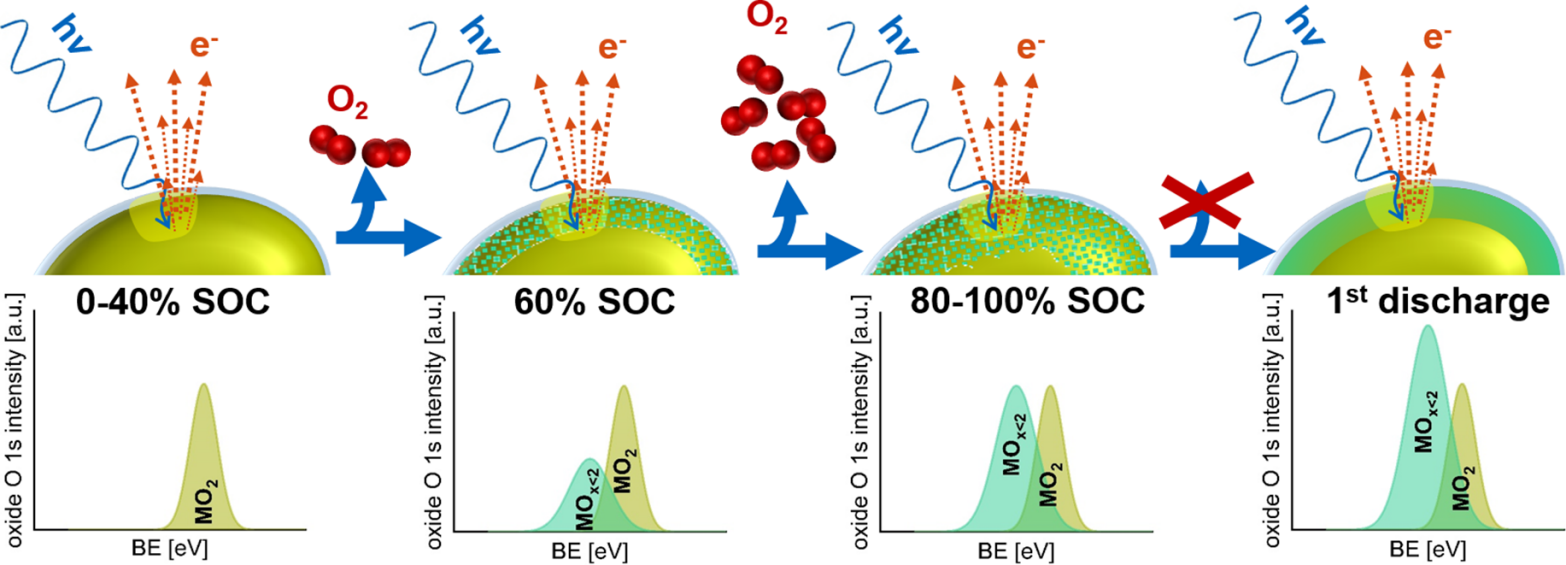
Schematic representation of the proposed steps of near surface lattice restructuring during the first charge/discharge cycle of NCM111 upon oxygen evolution based on the *operando* XPS data and the OEMS gas evolution analysis.

After its first emergence at 60% SOC (spectra 4), the relative amount of the formed *M*O_*x*<2_ phase (blue circles in Figure 5, right axis) increases continuously until the cell has been discharged to 45% SOC (55% SOD, spectra 9). At the end of charge (spectra 6), the relative *M*O_*x*<2_ share is 1.4 (based on a layered attenuation model corresponding to a thickness of roughly 6.2–6.5 nm of the oxygen-depleted phase, see the Supporting Information, section S5). Although the oxygen evolution as shown in Figure 2 is limited to the delithitation half-cycle, the amount of probed oxygen-depleted phase still increases by 35% during discharge to a final *M*O_*x*<2_ share of 1.9 (based on a layered attenuation model corresponding to a thickness of roughly 7.5–7.7 nm; for discussion of the oxygen-depleted layer thickness, see the Supporting Information, section S5). We assume that this increase is not caused by continuation of oxygen removal during the discharge of the *operando* APXPS measurement but rather by intraparticular restructuring as explained later. A schematic of the proposed restructuring during the first cycle of the NCM111 material in the battery MEA configuration can be found in Figure 6.

By using the *M*O_*x*<2_:*M*O_2_ ratio and absolute intensity of the Ni 2p_3/2_ spectrum, one can calculate the overall *M*:O ratio in the probed fraction of the NCM111 material, which is displayed as filled green squares in Figure 5. Details of this calculation can be found in the Supporting Information in section S4. During the first three sets of spectra during the *operando* APXPS measurement (0% SOC, 20% SOC, and 40% SOC) before the emergence of the oxygen-depleted phase at 60% SOC, the *M*:O ratio is at 0.5, which is expected for a layered oxide. From this point on, the *M*:O ratio increases continuously until a final value of 0.65 is reached, directly indicating that the formation of the additional feature in the O 1s spectra does indeed represent an oxygen-depleted phase. If the feature actually arose from parasitic reactions of the polymer or solvent, the *M*:O ratio shown in Figure 5 would need to decrease (as we would have wrongfully ascribed a component without *M* to the oxide phase). In case this additional component in the O 1s would stem from a surface near oxidation of the O^2–^ in the crystal lattice, as suggested in the literature,^56,57^ the *M*:O ratio would remain constant throughout cycling and this feature would need to decrease in intensity and eventually vanish during discharge.

For comparison, one can calculate the *M*:O ratio assuming that the oxygen-depleted phase is either a spinel or a rock-salt structure (*M*_3_O_4_ or *M*O, hollow dark blue or blue squares in Figure 5, left axis, see the Supporting Information, section S5 for detailed explanation). Although the experimentally observed *M*:O ratio for all spectra is closer to the expected value for the formation of a spinel than a rock-salt structure, the low intensity of the Ni 2p_3/2_ spectra leads to a large error range in determining the *M*:O ratio. While these results indicate that a spinel type of structure is probably formed after oxygen depletion from NCM111, the formation of a rock-salt—or even the combination of both—cannot be ruled out completely. The formation of a spinel-like structure for layered transition metal oxide cathode active materials with comparably low nickel content has been observed in literature using standard techniques as X-ray diffraction and transmission electron microscopy,^15−19^ which supports our findings for the studied NCM111 material.

The proposed steps of near-surface lattice restructuring upon oxygen depletion of the NCM111 material during the first charge and consecutive discharge are shown schematically in Figure 6, combining findings from both the *operando* XPS data and OEMS gassing analysis of the NCM battery MEA.

Below 60% SOC, no oxygen gassing could be observed, and consistently no oxygen-depleted *M*O_*x*<2_ phase features were detected in the *operando* XPS O 1s data set. Some oxygen evolution was observed via OEMS below 80% SOC, a little earlier than for standard electrode configurations, and it coincides with the appearance of the oxygen-depleted *M*O_*x*<2_ phase feature in the *operando* XPS O 1s spectrum. The majority of the oxygen evolution from NCM111 is observed between 80% SOC and 100% SOC, based on the OEMS gas evolution analysis. As the XPS probing depth is limited to the outermost layers, the XPS signal of the oxygen-depleted phase increases only slightly, however.

Although oxygen evolution is limited to the charging cycle of the material, the signal of the oxygen-depleted phase in the O 1s XPS spectra keeps increasing slightly until the end of discharge. This can be explained by structural rearrangement within the particle, leading to two locally constrained phases. Defects in the crystal structure may enable the evolution of oxygen from parts of the material located deeper in the primary particle than the XPS can probe, leading to a poorly structured “thick” layer of slightly oxygen-depleted phase. Oxygen migrates from the surface of the particle toward the bulk to stabilize the overall particle crystal structure into two spatially constrained phases, the original layered oxide structure and a more structured oxygen-depleted phase that forms predominantly on the particles’ surface. Therefore, the relative amount of *M*O_*x*<2_ in the probed fraction of the NCM material increases. We hypothesize this restructuring to be time-dependent—based on the rather linear increase in *M*O_*x*<2_ share observed in the XPS spectra after oxygen evolution—and not (predominantly) influenced by the relithiation of the material.

Combining OEMS gas evolution analysis data with the information gained by *operando* XPS measurements yields a deeper understanding of the step-wise restructuring of the layered transition metal oxide material throughout the first cycle. The observed changes in the O 1s spectra are providing additional information to *ex-situ* soft XAS studies in literature that have shown the surface near reduction of the transition metals upon oxygen evolution,^20,21^ which is linked to the oxygen-depleted phase formation tracked here. Time-dependent steps as the confinement of the oxygen-depleted phase on the particle surface after oxygen evolution emphasize the importance of developing new *operando* techniques to leverage information gathered by *ex-situ* data and to fully understand changes in interfaces and interphases of electrochemical systems during operation.

## Conclusions

In this study, we have shown that the *operando* monitoring of oxygen depletion from layered oxide cathode active materials for Li-ion batteries is possible by using the surface-sensitive XPS technique. As a result, a new MEA battery cell design has been developed for *operando* Li-ion battery material analysis at reduced pressures that shows minimal electrochemical differences from standard liquid electrolyte cells and could be an approach for studying such materials with techniques that require both high vacuum and direct access to the active material surface. By controlling the film thickness of the electrolyte by using a polymer electrolyte, measurements taken at different spots and different SOC steps can be compared quantitatively. We showed that an oxygen-depleted phase is formed upon first delithiation of the NCM111 material at high SOC and that it prevails throughout discharge. By comparing the relative intensities of the layered oxide, the oxygen-depleted phase, and the nickel in the mixed oxide, we demonstrated the expected correlation of oxygen depletion to an increase of transition metal-to-oxygen ratio. Although a rock-salt structure cannot be completely ruled out, the experimental transition metal-to-oxygen ratio instead indicates the formation of a spinel-like stoichiometry. Such new analysis techniques will be key to understanding the evolution of near-surface composition of Li-ion battery materials during cycling.

## Supporting Information Available

S1: evaluation of the ionic conductivity of the polymer electrolyte upon soaking with propylene carbonate and symmetrical Li-pump experiment for overpotential determination, S2: discussion of electrical conductivity and SOC inhomogeneity of the NCM111 battery MEA electrode, S3: estimation of the swollen polymer film thickness and XPS probing depth, S4: XPS reference measurement and peak assignment in the O 1s spectra, S5: details on fitting of *operando* XPS spectra and the full O 1s *operando* data set including estimation of oxygen-depleted phase thickness and the *M*:O ratio, S6: outlook on beam-induced damage of the PFSA-based polymer electrolyte and anodic oxidation study, S7: OEMS measurement and further additional experimental methods, and S8: comparison of *ex-situ* and *operando* O 1s XPS characteristics (PDF)

## ABBREVIATIONS

APXPS: ambient-pressure X-ray photoelectron spectroscopy
BE: binding energy
EDX: energy-dispersive X-ray spectroscopy
IPA: iso-propanol
MEA: membrane electrode assembly
OCV: open-circuit voltage
OEMS: on-line electrochemical mass spectrometry
PC: propylene carbonate
PFSA: perfluorosulfonic acid
SEM: scanning electron microscopy
SOC: state-of-charge
SOD: state-of-discharge
