# Supplementary material for: Surface Oxygen Depletion of Layered Transition Metal Oxides in Li-Ion Batteries Studied by Operando Ambient Pressure X-ray Photoelectron Spectroscopy
Source: ACS Appl Mater Interfaces. 2023 Jan 9;15(3):4743–54. doi: 10.1021/acsami.2c19008 (PMC9880953; doi:10.1021/acsami.2c19008)
Supplement: Supplementary file 1 — am2c19008_si_001.pdf [file am2c19008_si_001.pdf]

Supporting Information:

Surface Oxygen-Depletion of Layered Transition Metal Oxides in Li-Ion Batteries Studied by *Operando* Ambient Pressure X-ray Photoelectron Spectroscopy

Anna T.S. Freiberg[1†], Simon Qian[1], Johannes Wandt[1], Hubert A. Gasteiger[1*], Ethan J. Crumlin[2,3*]*

[1] Chair of Technical Electrochemistry, Department of Chemistry and Catalysis Research Center, Technical University of Munich, D-85748 Garching bei München, Germany

[2] Advanced Light Source, Lawrence Berkeley National Laboratory, Berkeley, California, 94720, United States

[3] Chemical Sciences Division, Lawrence Berkeley National Laboratory, Berkeley, California, 94720, United States

* a.freiberg@fz-juelich.de * ejcrumlin@lbl.gov * hubert.gasteiger@tum.de

S1. Ionic Conductivity and Overpotential of the Battery MEA

S2. Electrical Conductivity and Inhomogeneity Discussion for the Battery MEA

S3. Polymer Swelling and Layer Thickness Estimate

S4. XPS References for the Peak Assignment of the *Operando* Spectra

S5. Fitting of *Operando* O 1s XPS Spectra and Full *Operando* Data Set

S6. Beam-Induced Damage and Anodic Decomposition of the Polymer Electrolyte

S7. OEMS Measurements and Additional Experimental Methods

S8. Comparison of *Ex-situ* and *Operando* O 1s Spectra

S1. Ionic Conductivity and Overpotential of the Battery MEA

In order to achieve maximum conductivity of the Li-ion exchanged polymer membrane and LITHion^{TM} ionomer in the electrodes, the solvent uptake behavior and corresponding conductivity after exposure to propylene carbonate (PC) solvent was studied carefully. Samples of the Li-ion exchanged membrane were either i) directly immersed in excess of the PC solvent, ii) placed in the *operando* XPS cell with the PC reservoir in the glass fiber rings located below the membrane, iii) stored in a closed beaker over PC vapor (without direct contact to the liquid phase), or iv) simply stored in the glovebox (GB), where other organic vapors might influence the conductivity. After certain timespans under the above conditions, the membrane samples were weighed to determine the amount of PC absorbed. After soaking for 12 h they were characterized by electrochemical impedance spectroscopy (EIS) in the *operando* XPS cell in the GB, whereas a polished, closed stainless steel lid was used instead of the lid with the milled conical slit for *operando* XPS measurements. For the membrane soaked in excess of PC, excess solvent was removed by placing the sample on a clean-room wipe. A potentiostatic EIS with a perturbation amplitude of 10 mV in the range of 200 kHz to 10 Hz was executed to get the high-frequency resistance after 12 h of exposure, from which the conductivity of the membrane can be calculated.

The weight-uptake of the membranes and the conductivities obtained are shown in **Figure S 1**a. Three membranes were soaked for each system and the average value and standard-deviation is shown. In addition to the pure weight uptake in % $\left(\frac{m_{w/PC}-m_{dry}}{m_{dry}}\right)$on the left axis, the λ-value $\left(\frac{mol_{PC}}{mol_{SO_3}}\right)$ is shown on the right axis as it is a common way of expressing solvent uptake in PFSA based membranes.

When directly immersed in excess PC solvent, the weight-uptake is roughly 66%$_{wt}$, or $\lambda = 7.1\ \frac{mol_{PC}}{mol_{SO_3}}$, resulting in a conductivity of 0.02 mS/cm (see orange bar in **Figure S 1**). These values are constant over time and in good agreement with the study by Doyle et al.[1] The membrane stored in the GB atmosphere (black line) shows hardly any weight-gain over time and stays at a conductivity of less than 1 µS/cm. Storing the membrane over PC vapor, the weight uptake is really slow: after 12 h, the amount of PC absorbed in the membrane samples is roughly 10%$_{wt}$ and the conductivity is still only 3 µS/cm (blue line). On the other hand, when the membrane is contacted to a pool of PC solvent as done in the *operando* XPS cell, the weight-gain of PC in the membrane is fast and basically in equilibrium after 12 h, with a conductivity of 0.018 mS/cm (green line). Even after more than 3 days in the *operando* XPS cell stored in the argon-filled GB, the membrane shows basically no additional liquid film on the surface and the weight-uptake is finalized around 66%$_{wt}$, as is the case for the membrane soaked in excess PC solvent. This soaking procedure is adopted furtheron to allow cycling of the battery membrane electrode assembly (MEA) after short time of soaking, while still carefully controlling the PC uptake and omitting any additional liquid film formation. For the NCM111 MEA a small amount of PC (20 µl) is directly given onto the NCM111 working electrode to ensure good ionic conductivity. This droplet is completely absorbed by the electrode after 1 h.

In order to examine the electrochemistry of the overall battery MEA approach, a lithium pumping experiment was executed. Pristine LFP electrodes were cycled vs the partially delithiated $Li_{0.1}FePO_4$ and the polarization was recorded when cycling the electrodes between 15% and 85% state-of-charge (SOC) each. The current density was adjusted to represent the currents for the

NCM working electrode cycling at C-rates of $C_{NCM}/30$, $C_{NCM}/20$ and $C_{NCM}/10$, i.e., with 93 µA/cm² corresponding to $C_{NCM}/10$ based on an average loading of $3.4 \frac{mg_{NCM}}{cm^2}$.

The polarization of the symmetrical LFP battery MEA set-up as shown in **Figure S 1**b is highly symmetric and directly proportional to the current density. Roughly twice the capacity that is needed to fully cycle the NCM working electrode can be provided by the $Li_{0.1}FePO_4$ counter electrode. Based on these results we estimate the overpotential of the counter electrode and membrane for the NCM battery MEA to be on the range of 300-600 mV during cycling at C/10.

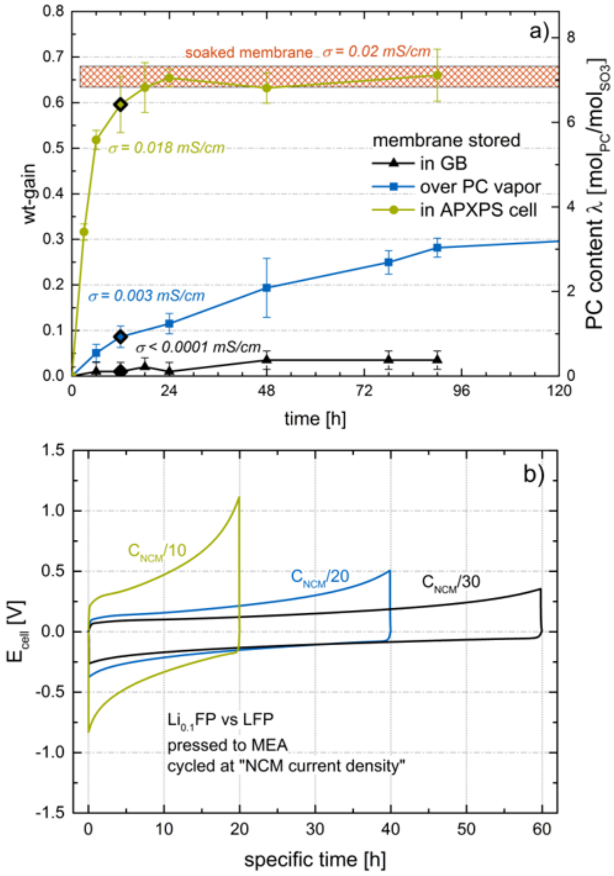

Figure S 1: Electrochemical characterization of the Li^+-conducting membrane and of the LFP counter electrode. a) PC content and ionic conductivity of the Li-ion exchanged Nafion™ HP membrane after 12 h under different PC solvent exposure conditions. b) Electrochemical response of the $Li_{0.1}FePO_4$ counter electrode cycled vs $Li_{1.0}FePO_4$ at current densities corresponding to C/10, C/20 and C/30 for the NCM working electrode.

S2. Electrical Conductivity and Inhomogeneity Discussion for the Battery MEA

Parts of the NCM111 working electrode have to be kept open to the XPS chamber in the *operando* XPS cell set-up in order to perform XPS analysis. It is therefore crucial to test whether the area that is probed and electrically connected only via the *in-plane* conductivity of the electrode to the current collector plate is representative of the SOC of the whole electrode. In order to study this, the *operando* XPS cell without the milled conically slit in the top plate is used. To achieve different areal ratios of the area directly connected to the current collector plate to area left open, a stainless steel disk (1 mm thickness) in different geometries is used and placed between the NCM111 working electrode and the current collector plate as shown in the scheme in **Figure S 2**. Starting from a full disk with a diameter of 15 mm (i.e., the same size as the working electrode area), a hole was drilled into the center of the disk with increasing diameter, therewith increasing the share of active material that can only be electrochemically cycled if the electrical *in-plane* conductivity of the electrode is sufficient. By choosing a diameter of the hole of 5 mm and 11 mm, the share of not directly connected NCM111 material accounts for 11% and 54%, respectively. The polarization of those 3 different configurations for cycling the battery MEA cell at C/10 with 20% SOC steps followed by OCV holds of 1 h (i.e., the intermittent C/10 cycling procedure used for the OEMS gassing analysis) is shown in **Figure S 2**.

Based on the overall polarization characteristics and the achieved charge and discharge capacity it is clear that the *in-plane* conductivity of the electrode is sufficient to allow cycling without obvious inhomogeneity caused by the area left open. In the *operando* XPS cell the slit milled into the top current collector plate to allow spectroscopy on the active material accounts for less than 9% of the electrode area (slit width of 2 mm), so we can expect the spectra to represent the state of the whole electrode *in-plane*.

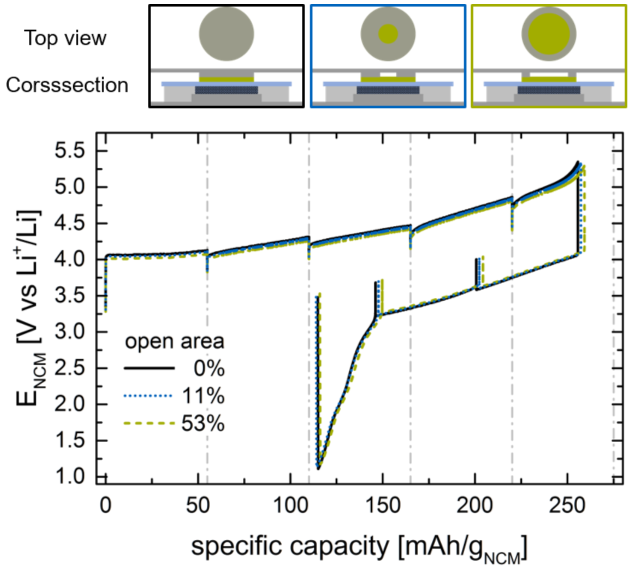

Figure S 2: Electrochemical response of the NCM111 battery MEA in dependency of area left open for spectroscopic analysis. The schemes on top depict the different configurations with full coverage of the NCM working electrode (black line and box), an inner open diameter of 5 mm (blue dotted line and box), and an inner open diameter of 11 mm (green dashed line and box).

Now that a sufficiently high electrical conductivity in the *in-plane* direction has been assured, the question arises as to why the O_2 release has been observed at an earlier average SOC and why also the oxygen-depleted phase emerged accordingly earlier in the XPS spectra. This will have to arise from an interparticular inhomogeneity in SOC, i.e., some particles of the NCM111 electrode reach the crucial SOC of 75-80% earlier because other particles have higher ionic or electrical resistances for lithium ion deintercalation. As the electrical *in-plane* conductivity has been studied already, the only remaining possibly limiting factors are the ionic and electrical conductivity in the *through-plane* direction. A depiction on how such limitations could change the distribution of SOC throughout the NCM111 electrode is shown in **Scheme S 1**.

Scheme S 1: Representation of expected distribution of SOC throughout the NCM111 electrode in case the electrical or ionic conductivity induces inhomogeneity with respect to SOC.

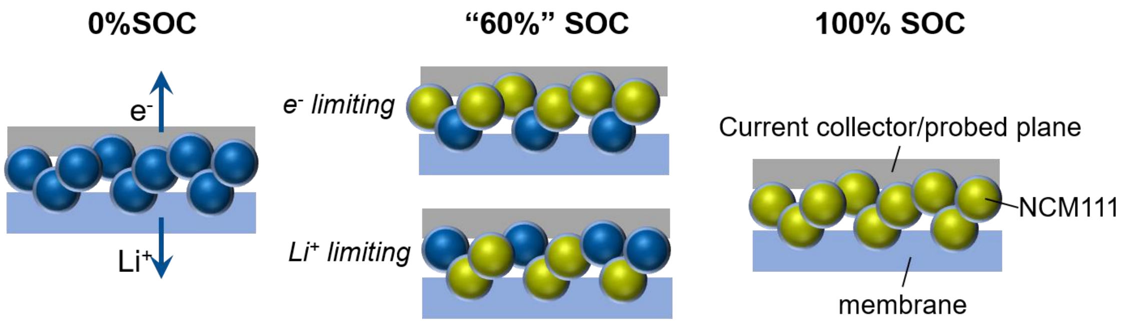

The gassing analysis does not differentiate whether the gas stems from the top of the NCM111 electrode (i.e., the side facing the current collector and probed plane) or from somewhere within; it just indicates that the SOC is inhomogeneous. The XPS result on the other hand shows that the top layer of the NCM particles, which is probed by the beam, is the region where the SOC rises faster than the SOC averaged over the whole electrode. Based on the fact that the ionic resistance is the highest for the top layer of the NCM electrode – as those particles are the furthest away from the membrane (Li^+ limiting) – it is clear that the reason for the inhomogeneity in SOC within the electrode has to stem from a limited electrical pathway to some of the NCM particles (see **Scheme S 1**). As we have shown in the afore section that the *in-plane* electrical conductivity of the NCM111 electrode is sufficiently good, the *through-plane* electrical conductivity seems to be the limiting factor, leading to a layer of relatively more delithiated NCM111 particles at the top of the electrode. In general, one would not expect an anisotropic electrical conductivity of the electrode. It could, however, be possible that the thin film of LITHion^{TM} polymer on top of the particles close to the membrane swells so much by the solvent delivered from the PC reservoir at the bottom of the cell that some particles lose some of their direct electrical connection to neighboring active material particles or the conductive carbon additive (e^- limiting).

S3. Polymer Swelling and Layer Thickness Estimate

It is known that the cationic conductivity of PFSA (perfluorosulfonic acid) –based ionomeric polymers as Nafion™ and LITHion™ is highly dependent on solvation and is far too low for electrochemical measurements in their dry state. Several solvents have been studied regarding their solvation effect and their effect on the Li-ion conductivity in PFSA ionomers.[1] In order to use the MEA in high vacuum, a solvent with low vapor pressure has to be chosen, which is why linear carbonates cannot be used. Ethylene carbonate on the other hand is solid at room temperature. Therefore, propylene carbonate is chosen as solvent to increase the ionic conductivity, as its vapor pressure of < 8 Pa at room temperature[2-3] is far below any other solvent commonly used in Li-ion batteries.

In order to control the analysis depth of the MEA, the film thickness of the polymer during operation has to be kept constant. The dry film thickness of the polymer can be calculated using the BET surface area of the NCM111 material $\left(A_{BET,NCM} = 0.23\,\frac{m^2}{g_{NCM}}\right)$ and the conductive carbon C65 $\left(A_{BET,C65} = 63\,\frac{m^2}{g_{C65}}\right)$, along with their relative weight share and the density of the dry PFSA polymer $\left(\rho_{Pol} = 1.93\,\frac{g}{cm^3_{Pol}}\right)$. From the BET area and the crystallographic density of NCM (ρ_{crystl}), one can estimate the active material to be best represented by spherical particles with a diameter d_{NCM} of 5.5 µm according to Equation 1:

$$d_{NCM} = \frac{6}{A_{BET,NCM}\cdot\rho_{crystl}} = \frac{6}{0.23\frac{m^2}{g_{NCM}}\cdot 4.75\cdot 10^6 \frac{g_{NCM}}{m^3}} = 5.5\,\mu m \qquad \text{(Eq 1)}$$

Based on the weight ratio of 90/6/4 of the NCM/C65/LITHion™ working electrode, the amount of the polymer on the surface of the C65 and NCM (l_{Pol}) can be calculated acc. to Equation 2:

$$l_{Pol} = \frac{m_{Pol}}{A} = \frac{0.04}{0.9 \cdot A_{BET,NCM} + 0.06 \cdot A_{BET,C65}} = 10\,\frac{mg_{Pol}}{m^2} \quad \text{(Eq 2)}$$

The surface film of dry polymer on a spherical NCM111 particle (t_{dry}, assuming a homogeneous layer covering the whole particle) would therewith correspond to

$$t_{dry} = \left[\sqrt[3]{\frac{l_{Pol} \cdot \left(\cdot 4 \cdot \pi \cdot \left(\frac{d_{NCM}}{2}\right)^2\right)}{\frac{4}{3}\pi \cdot \rho_{Pol}} + \left(\frac{d_{NCM}}{2}\right)^3}\right] - \frac{d_{NCM}}{2} = 5.2\,nm \quad \text{(Eq 3)}$$

The polymer shall be soaked with just enough PC solvent to reach optimum conductivity, without additional formation of a pure liquid film. We therefore examined the swelling behavior of Li^+-exchanged Nafion^{TM} in PC and measured the ionic conductivity by impedance spectroscopy as shown in section S1 of the SI. Based on those results, we decided on the soaking procedure explained in the Experimental section.

The weight-gain of the Li^+-exchanged Nafion^{TM} membrane in PC corresponds to roughly 66%$_{wt}$ as shown in section S1. For a thin film as in this case, the thickness increase by swelling can be approximated by a planar configuration (i.e., omitting the curved surface of the NCM particles) as given in Equation 4.

$$t_{wet} = t_{dry} \cdot \left(1 + \frac{0.66}{\frac{\rho_{PC}}{\rho_{Pol}}}\right) = 10.72\,nm \quad \text{(Eq 4)}$$

where ρ_{PC} is the density of PC and ρ_{Pol} is the density of the polymer in its dry state. While the attenuation length of X-rays at 5 keV in the active material is on the order of 5 µm (i.e., one whole secondary particle diameter), the inelastic mean free path (IMFP) of photoelectrons with a kinetic energy of roughly 4.47 keV (i.e., stemming from the O 1s orbital) is a good estimate for the actual

probing depth into the electrode. Using the *QUASES-IMFP calculation by TPP2m formula* software the IMFP of photoelectrons at 4.47 keV through the polymer electrolyte (PFSA+PC) can be estimated to be roughly 9.5 nm, whereas the IMFP of the active material is on the order of 7 nm. As the IMFP represents the distance a photoelectron can travel through a matrix with an intensity decay of 1/e, the active material below the polymer electrolyte film will still be probable. Furthermore, this close match of the estimated polymer thickness and the IMFP of the photoelectrons will result in the measured XPS data to truly represent the outer-most layer of the cathode active material from where oxygen is evolving. In order to estimate the layer thickness of the polymer electrolyte film covering the active material based on the *operando* APXPS data, we use the layered attenuation model as done by Etxebarria et al.[4] This model is based on a layered structure of several planar slaps of constant thickness overlaying. It can therefore only serve as a rough estimate minding the more complex structure the NCM111 working electrode consists of.

Assuming a homogenous overlayer of material B on a flat substrate A, the intensity of the detected photoelectrons of A I_A is reduced exponentially following Equation 5.

$$I_A = I_A^0 \cdot e^{-\frac{d_B}{\lambda_B \cos(\theta)}} \qquad \text{(Eq 5)}$$

Here d_B is the thickness of the overlayer B, λ_B is the IMFP of the respective photoelectron through B, and θ is the emission angle of photoelectrons with respect to the surface normal. I^0 refers to the intensity without attenuation of the overlayer. The intensity of photoelectrons generated from B follows

$$I_B = I_B^0 \cdot \left(1 - e^{-\frac{d_B}{\lambda_B \cos(\theta)}}\right) \qquad \text{(Eq 6).}$$

For the pristine electrode and at early SOCs, an estimate of the polymer electrolyte film thickness d_B=d_{ely} on top of the cathode active material can be executed, as the relative intensity of the O 1s spectra of the corresponding features are used, making the energy of the photoelectrons almost identical.

$$d_{ely} = \lambda_{ely} \cos(\theta) \ln\left(1 + \frac{I_{ely} I^0_{MO_2}}{I_{MO_2} I^0_{ely}}\right) \qquad \text{(Eq 7)}$$

Without attenuation, the ratio of the intensities of the substrate and the overlayer $\left(\frac{I^0_{MO_2}}{I^0_{ely}}\right)$ depends on the atomic density N and the IMFP of both materials with respect to the O 1s photoelectrons only.

The thickness of the polymer overlayer can therewith be calculated following Equation 8.

$$d_{ely} = \lambda_{ely} \cos(\theta) \ln\left(1 + \frac{I_{ely}}{I_{MO_2}} \cdot \frac{N_{MO_2} \cdot \lambda_{MO_2}}{N_{ely} \cdot \lambda_{ely}}\right) \qquad \text{(Eq 8)}$$

Because the intensity of the electrolyte based features in the O 1s spectra is only partially stemming from electrolyte covering the active material, the overall intensity I^{tot}_{ely} has to be corrected. Based on the approximation that the polymer film is spreading out to the same areal loading on the conductive carbon additive and the active material (see Equation 2), the absolute intensity observed has to be corrected based on Equation 9.

$$I_{ely} = I^{tot}_{ely} \cdot \frac{A_{NCM}}{A_{tot}} = I^{tot}_{ely} \cdot \frac{0.90 \cdot 0.23 \frac{m^2}{g}}{0.06 \cdot 63 \frac{m^2}{g} + 0.90 \cdot 0.23 \frac{m^2}{g}} = \frac{I^{tot}_{ely}}{19.26} \qquad \text{(Eq 9)}$$

The O 1s intensity ratio between the polymer electrolyte (I_{ely}^{tot}) and the metal oxide (either purely MO_2 for spectra 1-3, or the sum of MO_2+$MO_{x<2}$) stays 10.7±1.2 for all spectra except spectrum 3 (40% SOC, see **Figure S 6**). Assuming this variation to be caused by a change in film thickness covering the electrode material (active material and conductive carbon), the polymer electrolyte film thickness d_{ely} for these spectra can be estimated using the material parameters given in **Table S 1** to be 9.0±0.7 nm using Equation 8 and 9. This experimentally observed value is very close to the purely calculated value of 10.7 nm when using Equation 4.

The motivation to go to an MEA based system to study the oxygen-depletion from NCM-based cathode active materials was the controlled electrolyte thickness and therewith the controlled probing depth into the active material. This small variation of electrolyte film thickness observed in the *operando* APXPS data set shows that the probing depth into the active material is indeed essentially constant. Therefore, the evolution of the oxygen-depleted phase forming out of the layered oxide structure at high SOC can be studied by quantitatively comparing the signal of the metal oxide features in the O 1s spectra.

Table S 1: Material parameters used for the polymer electrolyte overlayer estimate. The IMFPs are calculated using the software *QUASES-IMFP calculation by TPP2m formula*. In our set-up, θ is roughly 10°.

Material	Bulk density [g/cm³]	N [10^{22} mol_O/cm^3]	$\lambda_{4.47\ keV}$ [nm]
$LiMnO_2$ – layered oxide	4.04	5.18	7.35
Propylene carbonate	1.20	2.12	9.55
Li^+-exchanged Nafion	1.98	0.54	9.37
PC-soaked Nafion	1.57	1.37	9.50

S4. XPS References for the Peak Assignment of the *Operando* Spectra

The NCM111 working electrode consists of multiple components with numerous spectral features in the XPS data. Several references were measured at Beamline 9.3.1 of the Advanced Light Source (ALS) at the Lawrence Berkeley National Laboratory to ensure proper interpretation of the spectra. The references were measured at an X-ray energy of 5 keV, and the pressure within the analysis chamber was 10 Torr (1333 Pa).

For studying the oxygen-depletion from the layered oxide material, the low binding energy (BE) region of the O 1s was used in the main study. Besides the pristine NCM111 powder, NiO (>99.99%, Sigma Aldrich) and the LNMO spinel ($LiNi_{0.5}Mn_{1.5}O_2$, BASF, Germany) were measured to get a rough estimate of the O 1s position of the oxygen-depleted phase. The O 1s spectra of these samples are shown in **Figure S 3**, whereas energy calibration was executed based on an energy of 284.8 eV in the C 1s of the adventitious carbon.

All samples show broad features between 531-534 eV BE due to surface impurities like hydroxides and carbonates.[5] Well separated from these is the metal oxide region at 528-531 eV.[6] The peak position for oxygen-depleted stoichiometries like spinel (blue line) and rock-salt (gray line) of 530.0 and 530.2 eV BE, respectively, are substantially higher compared to the layered oxide peak at 529.3 eV (green line). Not just the stoichiometry but also the order and crystallinity influence the exact peak position. Therefore, the exact position of the oxygen-depleted phase can not be determined by reference measurements, but it is expected to emerge roughly 0.6-1 eV higher in BE compared to the layered oxide feature and still far from any influence from surface impurities.

Besides the active material, the polymer and PC solvent also contribute to the O 1s spectra that are the focus of this study. Therefore, a dry membrane and a membrane soaked in PC were measured

as references as well. Though pure spectra of the PC solvent have not been obtained, the reference energies given by Maibach et al.[7] for PC itself are used for the following analysis.

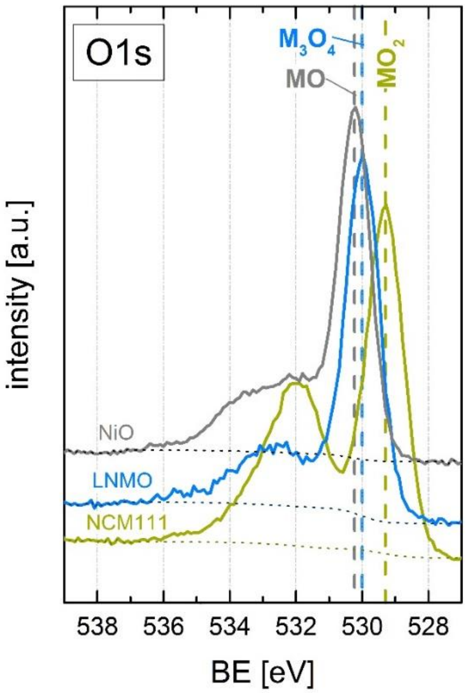

Figure S 3: O 1s spectra of transition metal oxide powder references. Layered oxide NCM111 (green line), spinel LNMO (blue line) and rock-salt NiO (gray line) are compared. The peak energy of the oxygen in the metal oxide of each sample is indicated by vertical dashed lines.

The left panel of **Figure S 4** shows the C 1s spectra of the dry (blue line) and soaked membrane (green line). A multicomponent fit was not executed due to the broad features at low BE. The predominant feature around 292.2 eV corresponds to the $–CF_2$ bonds of the polymer. Due to the low intensity of the adventitious carbon in the C 1s, the membrane references were energy calibrated to the $–CF_2$ value. In addition to this, there are $O-CF_2$ bonds within the polymer around 290 eV and single CF bonds around 287 eV,[8] which can however not be resolved properly due to the reduced atomic photoabsorption cross-section of carbon at 5 keV X-ray energy.

The membrane soaked in PC (green line in **Figure S 4**, left panel) shows two additional features at lower BE in the C 1s spectrum. Looking at the molecular structure of PC there are four differently hybridized carbon atoms: the carbon in the CH_3 side group, the carbon in the carbonate group only surrounded by oxygen and the two carbons in the ring that both bear one bond to an oxygen and at least one to a carbon atom. The latter two have been observed at a single energy of roughly 287 eV, the CH_3 carbon is expected around 285 eV, and the carbon only surrounded by oxygen atoms has a large shift to roughly 290.6 eV.[7] While the latter cannot be properly resolved in the C 1s spectrum of the PC soaked membrane due to the large CF_2 feature, the additional features at low BE fit very well with the published values for PC (black vertical dashed lines[7] in **Figure S 4**, left panel). This shows that the soaked membrane indeed contains PC that can be observed in XPS.

Checking the O 1s spectra of the dry and PC soaked membrane (**Figure S 4**, right panel), no significant difference in the spectra can be observed. The CF_2-O-CF_2 group can be seen around 533.9 eV and the $–SO_3$ is observed around 532.1 eV.[8] A two-component fit is able to approximate the dry and PC soaked membrane (see section S4 for details). Based on the values by Maibach et al.[7], PC should have two features in the O 1s spectrum, viz., the C=O oxygen around 534.1 eV and the C-O-C oxygen around 532.3 eV, which are indicated as black dashed vertical lines in **Figure S 4**. Both features are very close to the ones expected and observed for the dry polymer, which is why a differentiation between the polymer-based and solvent-based parts of the O 1s spectrum is not possible. The only change observed when soaking the membrane in PC is the sharpening of the peaks of the two-component fit. The dry membrane has a FWHM of the high BE and low BE component of 3.0 eV and 2.1 eV, which are sharpening to 2.2 eV and 1.5 eV in the PC soaked membrane, respectively. This sharpening can be explained by reduced inter-chain interaction

and/or ordering of the polymer chains[9] into hydrophobic backbone and hydrophilic side-chain regimes as has been observed for hydrated Nafion™.[10] Other broadening mechanisms like differences in conformation or defects could also be responsible for this observation.

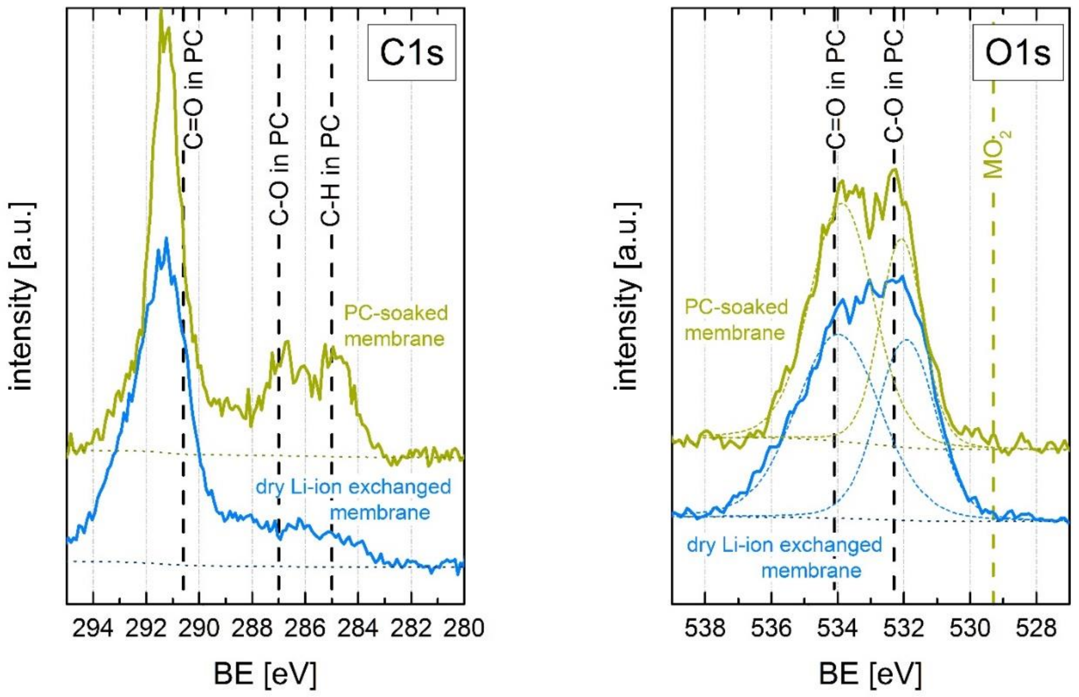

Figure S 4: C 1s and O 1s spectra of dry (blue) and PC-soaked membrane (green). In case of the O 1s, a two component fit is executed and indicated by dashed lines. Vertical dashed lines indicate peak positions found in the literature for PC and the position at which the metal oxide peak is located for the *operando* XPS measurements.

In order to get an estimate of the transition metal (*M*) to oxygen ratio, the Ni $2p_{3/2}$ region was quantified. The sensitivity at 5 keV toward *M* is significantly reduced, which is why only the Ni $2p_{3/2}$ was quantified, as it has the largest atomic photoabsorption cross-section of the three transition metals. As a quantification of the oxygen in the metal oxide, only the area below the lowest BE peak in the O 1s spectrum was taken for the reference compounds shown in **Figure S 3**, therewith omitting any overestimate caused by surface impurities as carbonates and hydroxides. For the Ni on the other hand the whole intensity of the corresponding Ni $2p_{3/2}$ spectrum was used.

In case nickel would be present as surface contaminant as in the form of nickel carbonate, this would lead to an overestimate of the relative sensitivity factor. The NCM111 material studied here is known to bear surface impurities in the form of carbonates, though mainly lithium carbonate was observed in contrast to Ni-rich layered oxide materials that are predominantly covered by nickel-bearing impurities.[5, 11] The relative sensitivity factor of the areas of the Ni $2p_{3/2}$ with respect to the O 1s peaks of the transition metal oxide was calculated based on the reference measurement of the NCM111 powder, resulting in $rsf_{Ni\ 2p_{3/2}}^{O\ 1s} \approx 3.9$. This reference-based relative sensitivity factor can also be obtained from the NiO and LNMO reference samples (i.e., 3.88 and 3.95, respectively) and is therefore seen to be a more reliable value to quantify the transition metal to oxygen ratio for the *operando* spectra compared to a theoretically calculated relative sensitivity factor. The fact that the relative sensitivity factors for the three different reference compounds are very close supports our approach of using the full area below the Ni $2p_{3/2}$ spectra, as we would expect different extents of surface contamination on the different materials and therewith a higher deviation of the calculated values in case nickel-bearing contaminants would have a significant impact on the nickel intensity. The Ni $2p_{3/2}$ of the NCM111 powder is shown in **Figure S 5** in the left panel. Energy calibration was executed based on the C 1s of the adventitious carbon at 284.8 eV.

The *operando* spectrum shown in the central and right panel in **Figure S 5** have a higher noise level and lower overall intensity. In order to account for the error in the quantification of the peak areas, different background limits were chosen and are shown in dark green lines. Therewith, an error range for the calculated *M*:O ratio is defined as shown in the main paper. The overall low sensitivity makes further analysis on the transition metal XPS spectra ambiguous and is therefore not executed.

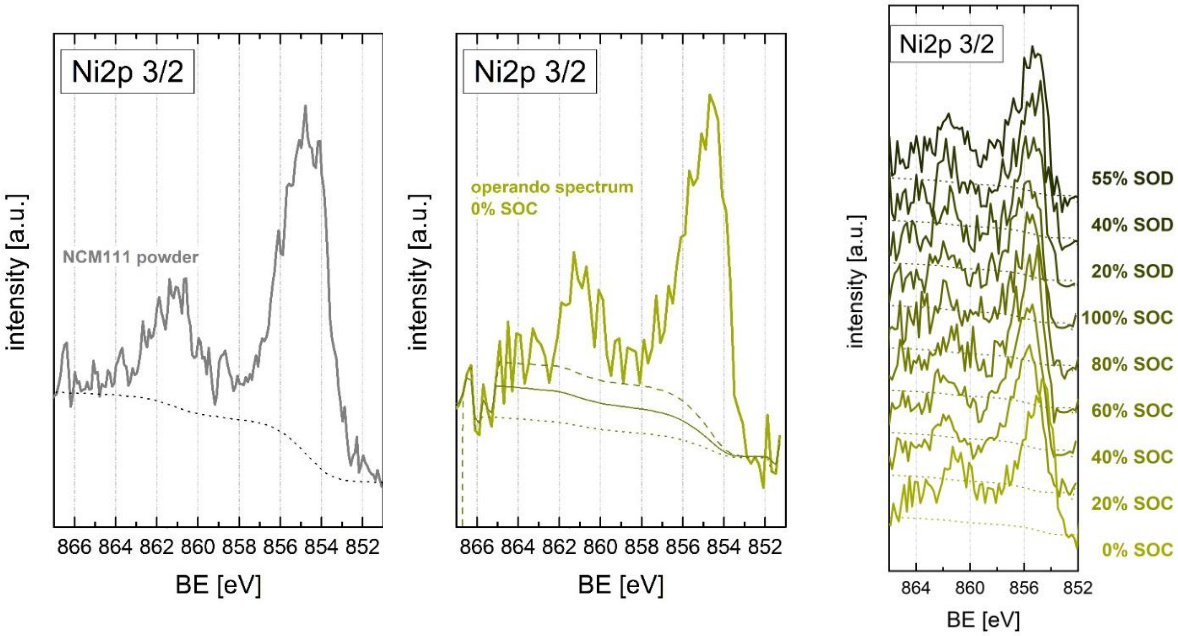

Figure S 5: Ni $2p_{3/2}$ spectra of the NCM111 powder reference (left panel) and exemplarily for the pristine spectrum (i.e., at 0% SOC) of the *operando* MEA (middle panel). In order to get an estimate on the error of the *M*:O ratio used in the main paper, different background limits were used. The normalized Ni $2p_{3/2}$ *operando* spectra for all recorded SOCs are shown with their average chosen background (right panel).

S5. Fitting of *Operando* O 1s XPS Spectra and Full *Operando* Data Set

The XPS spectra recorded were analyzed using the CasaXPS software package. Except for the membrane references (discussed in section S3 and this section), all spectra were energy calibrated to the C 1s of adventitious and/or conductive carbon at 284.8 eV BE. All spectra were analyzed using a Shirley background and the overall peak-shape of any component was fixed to a Gaussian-Lorentzian product form with 30% Gaussian character (GL(30)).

For the *operando* O 1s spectra most values were left open to not restrict the fit unnecessarily. The two high BE features of the electrolyte (polymer+PC) had no restrictions. The metal oxide peak corresponding to the layered MO_2 structure was only restricted to have a FWHM of less than 2 eV in order to ensure that the oxygen-depleted phase was not masked by a broadening of the MO_2 peak. In addition, this fit was forced to have a maximum variation in FWHM between spectra recorded at different SOC of ± 0.1 eV (i.e., the step size of the spectra). The oxygen-depleted $MO_{x<2}$ component was restricted to be at least 0.6 eV higher in BE than the MO_2 peak and to have a FWHM of less than 2 eV.

While the first spectra (0-40% SOC) contain only one peak in the metal oxide region assigned to the layered oxide phase, an additional peak was added to the fit for all other spectra, representing the oxygen-depleted phase. When looking carefully at the overall lineshape of the O 1s spectra around 530.5 eV, one could envision an additional small feature emerging between the layered oxide and the low BE electrolyte peak already at 20% SOC and 40% SOC. Adding one additional component to the fit, however, results in an ambiguous position of it. When leaving the position only restricted to be at least 0.6 eV higher in BE compared to the layered oxide feature (i.e., as done for all other spectra), the new component is optimized to sit around 531.8 eV, i.e., it tries to

improve the fitting quality of the broad low BE electrolyte feature. When adding an upper limit to the position restriction of this component, it will run into this limit when executing this fit. Furthermore, the relative intensity of this component becomes negligible (i.e., <3% of the layered oxide intensity in the O 1s) when approaching the position where the oxygen-depleted phase has been observed for the spectra obtained at higher SOC, as long as the FWHM stays restricted to a maximum of 2 eV. Adding this component already for the spectra obtained at low SOC is therefore not considered to be meaningful. This spectral shape change could be linked to the formation of electrolyte decomposition products that lead to a slight broadening of the electrolyte feature (see section S6). However, we do not want to completely exclude any observation of an oxygen-depleted phase in the *operando* XPS spectra at lower SOC, but we just want to stress the point that is unambiguously provided by the XPS data, which is the sudden, strong emergence of the oxygen-depleted phase around 60% SOC. A further understanding of the broad electrolyte features and their behavior upon polarization as well as a closer study on the evolution of surface impurities throughout cycling (as they also lay below the low BE electrolyte feature) would be necessary to go for a more detailed analysis of the spectra.

In the main paper only the metal oxide region of the O 1s is shown, as this is the region of interest. The full non-normalized O 1s spectra of the *operando* APXPS data set are shown in **Figure S 6**. The position and FWHM of all components in the O 1s spectra throughout cycling of the *operando* APXPS MEA are additionally given in **Table S 2**.

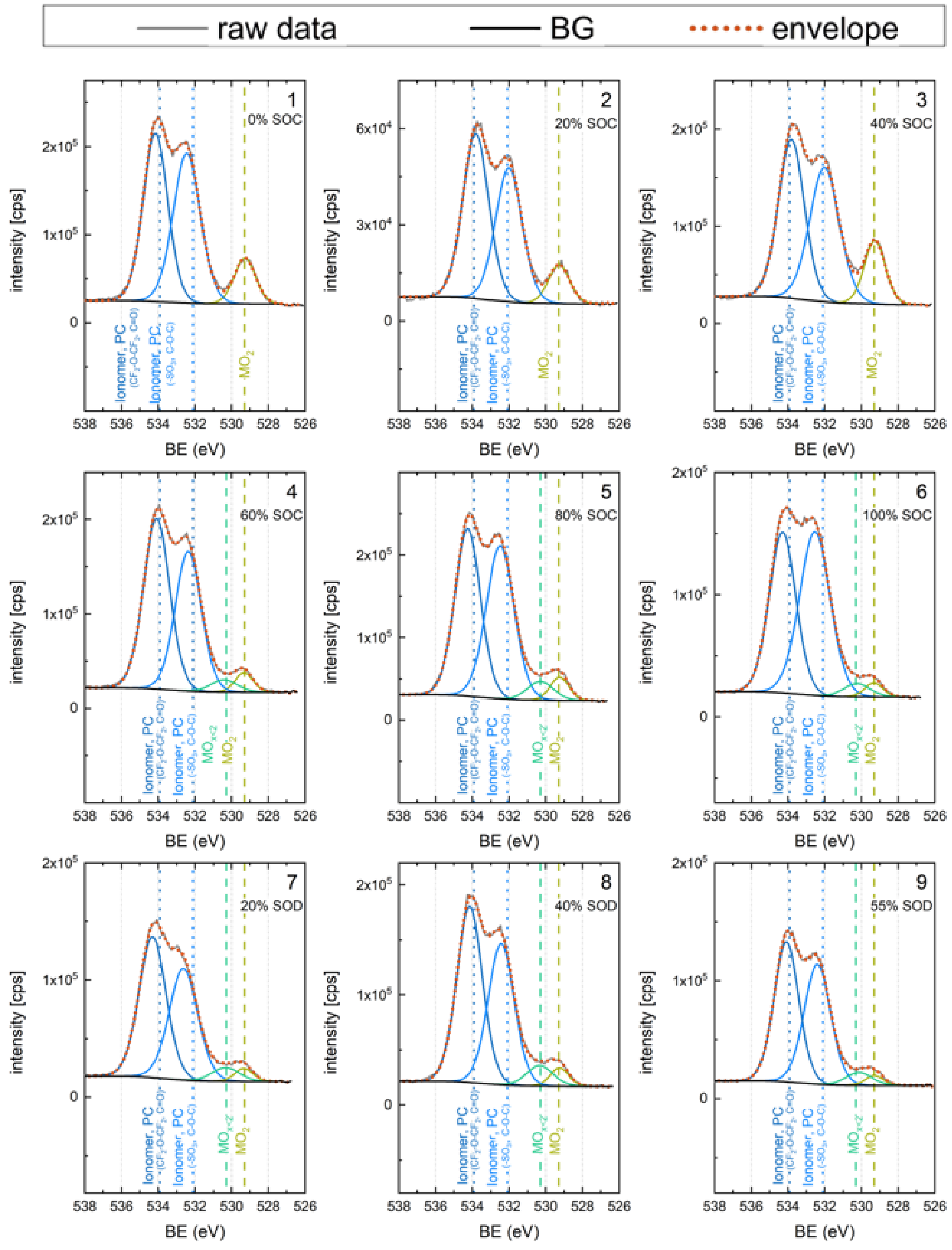

Figure S 6: Full O 1s spectra of the *operando* APXPS measurement of the NCM111 working electrode acquired for the C/10 cycling in 20% SOC steps with OCV holds. The afore discussed energies of electrolyte components are given in dotted blue lines and the expected energies for the metal oxides in dashed green and turquoise lines.

Table S 2: Position and FWHM of metal oxide and electrolyte components of the *operando* data shown in **Figure S 6** and fitted in a GL(30) lineshape over a Shirley background.

Spectrum	MO_2 feature		$MO_{x<2}$		Polymer, PC (-SO_3, C-O-C)		Polymer, PC (CF_2-O-CF_2, C=O)	
	BE [eV]	FWHM [eV]	BE [eV]	FWHM [eV]	BE [eV]	FWHM [eV]	BE [eV]	FWHM [eV]
1 – 0% SOC	529.27	1.35			532.44	1.74	534.15	1.57
2 – 20% SOC	529.27	1.35			532.01	1.70	533.81	1.66
3 – 40% SOC	529.26	1.35			532.01	1.90	533.81	1.65
4 – 60% SOC	529.32	1.15	530.40	1.70	532.36	1.66	534.08	1.67
5 – 80% SOC	529.25	1.19	530.30	1.69	532.49	1.78	534.25	1.63
6 – 100% SOC	529.32	1.15	530.24	1.68	532.54	1.96	534.29	1.69
7 – 20% SOD	529.27	1.15	530.22	1.73	532.54	1.87	534.22	1.71
8 – 40% SOD	529.25	1.15	530.23	1.70	532.33	1.67	534.05	1.63
9 – 55% SOD	529.29	1.16	530.13	1.67	532.40	1.70	534.08	1.57

The metal oxides peak of MO_2 at 529.3 eV BE and $MO_{x<2}$ at 530.3 are basically constant in position throughout the experiment (529.25-529.32 eV and 530.13-530.40 eV, respectively). This is expected, as the working electrode is grounded to the analyzer cone.

The polymer and solvent specific features around 532.1 eV BE and 533.9 eV BE, however, are slightly shifting. The peak position of the electrolyte-based peaks along with its basic energy measured by the *ex-situ* membrane references shown in **Figure S 4** and the electrochemical potential during which each spectrum was recorded is shown in **Figure S 7**.

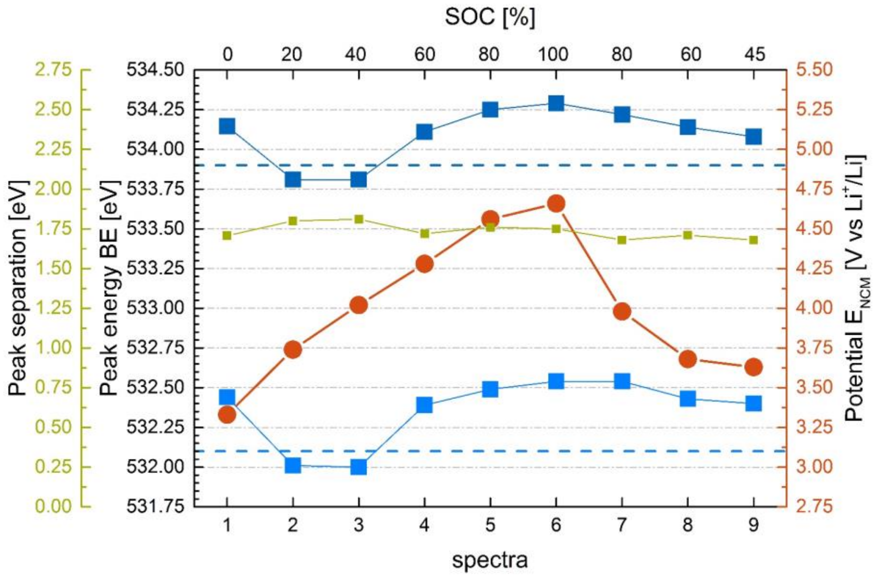

Figure S 7: Peak position (blue squares, inner left axis) and peak separation (green squares, outer left axis) of the two electrolyte components observed in the O 1s for all *operando* spectra shown in **Figure S 6**. Horizontal dashed lines indicate the peak position of the membrane *ex-situ* references. The OCV potential during which those spectra were recorded, is shown as orange circles on the right axis.

As shown in blue squares in **Figure S 7** (inner left axis), the peak position of both electrolyte-based features in the O 1s is shifting significantly for different points during cycling. The peak separation of both, however, (green squares, other left axis) is basically constant, which is required as both stem from the polymer and solvent in the electrolyte and should therewith bear the same relative shift caused by their electrically insulating nature. The exact position of the electrolyte-based features is not following the potential at which the O 1s spectra were recorded. As the electrical conductivity of these components is very low and as the majority of the electrolyte contributing to the signal in the O 1s spectra is far from the double-layer of the electrode, a shift of the peak energy in dependency of the applied electrochemical potential can be expected. The spectra were recorded during the open circuit voltage (OCV) period in the electrochemical cycling procedure, still the systems potential is different for each spectrum. O 1s spectra were recorded in sets of 10 scans that were averaged in order to obtain a good signal to noise ratio. We could observe

that the peak position of the electrolyte-based features at high BE is continuously shifting in between scans to higher BE either due to continuous relaxation of the OCV potential over time or due to beam effects. In addition, the electrolyte-based features in the O 1s spectra were only fitted with two components though they are in reality composed of 4 major components (2 for the Li-ion exchanged PFSA and 2 for the PC solvent) and possibly several smaller components caused by material impurities.

This reduced complexity in the fit of the electrolyte dominated region in the O 1s was chosen as the metal oxide region of interest is separated enough for the electrolyte fit to have no significant impact on the actually quantified components in the low BE region. A more thorough understanding of the evolution of the electrolyte-based features in the O 1s would require better signal to noise already within one scan and ideally concurrent tracking of the evolution of the C 1s and S 1s region. Future experiments will work to resolve these components and track the changes with potential.

The fitting parameters for the electrolyte components were not restricted. As the peak separation and areal ratio of both peaks are basically constant throughout all *operando* spectra (1.75±0.06 eV BE and 0.9±0.07, respectively), we conclude that both peaks are indeed dominated by the polymer and electrolyte features. Minding the change in FWHM observed for both features (see **Table S 2**), we assume that a differentiation of the PFSA- and PC-based electrolyte component would be beneficial for the overall fit quality but is not available for this set of data.

The *operando* data shows a continuous rise of the signal assigned to the formation of the oxygen-depleted metal oxide feature around 530.3 eV BE after exceeding roughly 60% SOC. Assuming the formation of an oxygen-depleted layer on the outside of the particle slowly growing thicker,

the thickness of this layer can be calculated using the same formulas used in section S3, based on the intensity ratio of the oxygen-depleted phase and the layered oxide structure. As we hypothesized in the main text, the geometrical restriction of the oxygen-depleted phase to the surface of the particle may only happen over time, but an estimate on the thickness of this oxygen depleted phase based on the intensity ratio observed can still be helpful in envisioning the restructuring process based on the *operando* APXPS data set. Please note that this estimate is again based on a layered attenuation model and, while it cannot represent the complex structure of the NCM111 working electrode, it shall be the base of a rough layer thickness estimate.

Though a mixed transition metal oxide is used in this study, the estimate on probing depth is executed using the material parameters for pure manganese oxides due to a lack of reported physical parameters for the mixed oxides. The layer thickness was estimated for either the formation of a spinel or a rock-salt phase. **Table S 3** summarizes the material parameters used:

Table S 3: Material parameters used for overlayer and penetration depth estimate. The IMFPs are calculated using the software *QUASES-IMFP calculation by TPP2m formula*. In our set-up, θ is roughly 10°.

Material	Bulk density [g/cm³]	N [10^{22} mol_O/cm^3]	$\lambda_{4.47\ keV}$ [nm]
$LiMnO_2$ – layered oxide	4.04	5.18	7.35
Mn_3O_4 – spinel	4.86	5.12	6.61
MO – rock-salt	5.45	4.63	6.30

In our experimental set-up the emission angle of photoelectrons with respect to the surface normal θ is roughly 10°. Based on Equations 5 and 6, the estimated thickness of the oxygen depleted phase can be calculated for the *operando* data sets 4-9 using:

$$d_{MO_{x<2}} = \lambda_{MO_{x<2}} \cos(\theta) \ln\left(1 + \frac{I_{MO_{x<2}}}{I_{MO_2}} \cdot \frac{N_{MO_2} \cdot \lambda_{MO_2}}{N_{MO_{x<2}} \cdot \lambda_{MO_{x<2}}}\right) \quad \text{(Eq 10)}$$

The obtained values for $d_{MO_{x<2}}$ are summarized in **Table S 4**

Table S 4: Estimated film thickness after surface oxygen depletion for the *operando* APXPS data, assuming either spinel or rock-salt formation.

O 1s spectrum	Areal ratio $\frac{I_{MO_{x<2}}}{I_{MO_2}}$	M_3O_4 spinel formation	MO rock-salt formation
		$d_{MO_{x<2}}$ [nm]	$d_{MO_{x<2}}$ [nm]
4 – 60% SOC	1.03	5.0	5.3
5 – 80% SOC	1.14	5.4	5.7
6 – 100% SOC	1.41	6.2	6.5
7 – 20% SOD	1.49	6.4	6.7
8 – 40% SOD	1.73	7.0	7.3
9 – 55% SOD	1.90	7.5	7.7

In order to obtain these values, the approximation of a flat surface with a homogeneous overlayer of oxygen-depleted phase on a layered substrate had to be used. It is known that particle cracking occurs during delithiation of polycrystalline NCM materials, which largely increases the surface area of the material.[6, 12-13] Oxygen release is believed to occur from the primary particle surface available after cracking, not exclusively from the outer surface of the secondary agglomerate. The approximation of a flat surface with a planar overlayer is therewith an upper limit of the oxygen-depleted phase layer thickness, as it forms on the surface of sub-micrometer sized primary crystallites. Additionally, due to the higher mass of nickel and cobalt compared to manganese, the approximation based on the physical parameters of pure manganese oxides is further seen as an overestimate of the thickness of the oxygen-depleted phase. Comparing the formation of a rock-

salt NiO – for which physical parameters are available – with the values obtained in **Table S 4** for the formation of MnO, the film thickness calculated decreases by roughly 18%.

Nevertheless, the values obtained in **Table S 4** are a reasonable estimate for the order of magnitude of surface layer formation of the NCM material upon delithiation to high SOC, and agree with the values obtained by X-ray diffraction, transmission electron microscopy, and electron diffraction of such materials, even though only severely aged NCM material could be studied with respect to their surface chemistry with such techniques, as explained in the introduction of the main text.

While the position of the peak in the O 1s corresponding to the oxygen depleted phase does not allow differentiation between spinel and rock-salt structure, the experimentally obtained $MO_{x<2}/MO_2$ (i.e., the area below the oxygen-depleted oxide peak around 530.3 eV in the O 1s spectra divided by the area below the layered oxide peak around 529.3 eV) allows an estimate on the possible stoichiometry of the oxygen-depleted phase formed. One can therewith calculate the theoretical *M*:O ratio (atomic ratio of transition metal to oxide oxygen) and compare it to the experimentally obtained *M*:O ratio as shown in **Figure 5** of the main text.

The $MO_{x<2}/MO_2$ ratio is solely based on the O 1s spectra and represents how much of the oxide phase probed by the beam is oxygen-depleted and how much is still present in the pristine layered phase. The stoichiometry coefficient in the three different considered phases of layered oxide, spinel and rock-salt are $x^{lo} = 2$, $x^{sp} = \frac{4}{3}$ and $x^{rs} = 1$, respectively. The *M*:O ratio as shown in **Figure 5** of the main text is simply $\frac{1}{x}$ and represent the molar ratio of transition metal present in the oxide structure (both depleted and layered oxide phase) to the overall amount of oxide oxygen. The total *M*:O ratio after oxygen depletion as a mixture of both the pristine layered oxide

stoichiometry x^{lo} and the oxygen-depleted phase stoichiometry x^{dep} can be calculated using Equation 11:

$$M:O = \frac{1}{x} = \frac{n_{Ni\,2p_{3/2}}^{MO_{x<2}} + n_{Ni\,2p_{3/2}}^{MO_2}}{n_{O\,1s}^{MO_{x<2}} + n_{O1s}^{MO_2}} = \frac{n_{O1s}^{MO_2} \cdot \frac{1}{x^{dep}} \cdot z + n_{O1s}^{MO_2} \cdot \frac{1}{x^{lo}}}{n_{O1s}^{MO_2} \cdot (1+z)} = \frac{\frac{1}{x^{dep}} \cdot z + \frac{1}{x^{lo}}}{(1+z)} \qquad \text{(Eq. 11)}$$

with $$\frac{MO_{x<2}}{MO_2} = z = \frac{n_{O\,1s}^{MO_{x<2}}}{n_{O1s}^{MO_2}}$$

Subscripts indicate the element of interest and corresponding orbital used for quantification of spectral intensity and superscripts indicate the phase of the element. This calculation does not rely on the relative sensitivity factor of the metal to oxygen intensity; as the theoretical stroichiometries of the depleted phases are known, the measured relative intensity of the two oxide phases (pristine layered and oxygen-depleted) based on their O 1s intensity can be converted into an expected *M*:O ratio in case of spinel ($x^{dep} = x^{sp}$) or rock-salt formation ($x^{dep} = x^{rs}$). This value can then be compared to the experimentally observed *M*:O ratio as executed in **Figure 5** of the main text.

S6. Beam-Induced Damage and Anodic Oxidation of the Polymer Electrolyte

Beam-induced damage is something that is frequently observed during X-ray based analysis. Especially when synchrotron radiation is used, the beam-induced damage can be severe and might impede investigation of certain systems completely. We therefore carefully examined any change in the O 1s spectra of an *operando* MEA at OCV during continuous irradiation of a single spot of the electrode.

As shown in **Figure S 8**, a significant change in the O 1s spectrum of the MEA exposed to PC can be observed over a time span of 60 minutes. While the overall intensity is decreasing over prolonged irradiation, especially the metal oxide region is nearly completely vanishing. This loss in intensity of the active material based features is also observed for the Ni $2p_{3/2}$ spectra, wherefore it is assumed that parts of the polymer electrolyte get decomposed upon irradiation and form an additional layer that hinders detection of photoelectrons from the active material. One possible mechanism would be the polymerization of PC from the gas phase on top of the electrode.

To better understand what is happening with the polymer structure upon irradiation, we executed a series of measurements of the membrane references shown in **Figure S 4** for the change in the C 1s, O 1s, F 1s, and S 1s region over prolonged irradiation. The results are mainly independent on whether the membrane is soaked with PC or not, the presence of PC just seems to slow down the process probably due to partial absorbance of the X-ray energy and associated heat and/or due to polymerization of PC on top of the electrode that would hinder further analysis of the polymer specific features.

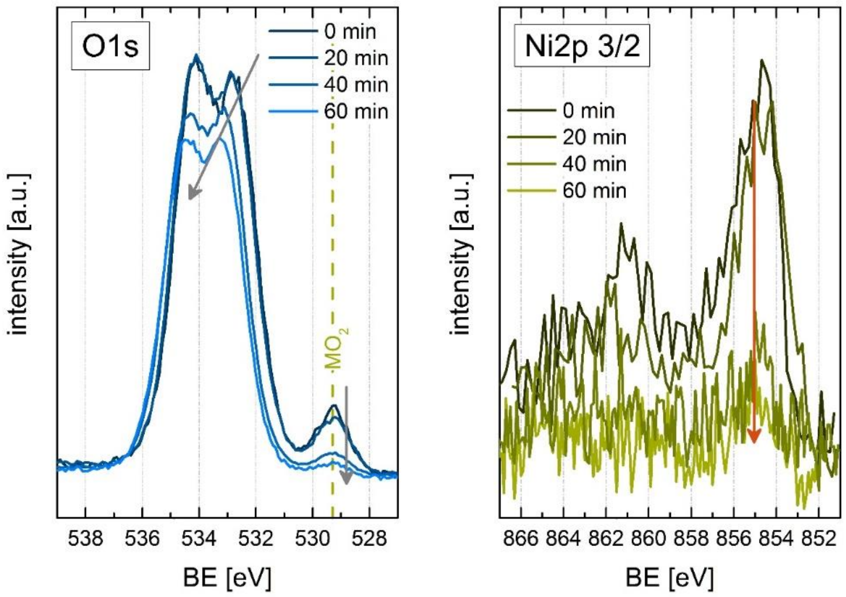

Figure S 8: Change of O 1s and Ni $2p_{3/2}$ signals of the NCM111 battery MEA with PC solvent during irradiation of a single spot over 60 minutes with 5 keV X-ray energy.

Figure S 9 shows the change in the S 1s, F 1s, O 1s and C 1s (from left to right) of the polymer without the PC solvent upon irradiation for more than 5 h. The raw intensity is plotted with a fixed off-set for each set of spectra. The F 1s spectra are divided by ten in intensity to show all spectra combined on a single scale. Energy calibration was executed based on the F 1s of the -CF_2 group in the pristine polymer. While the F 1s and O 1s spectra show only minor changes in peak intensity and sharpness, the S 1s and C 1s spectra change significantly with respect to their chemical information.

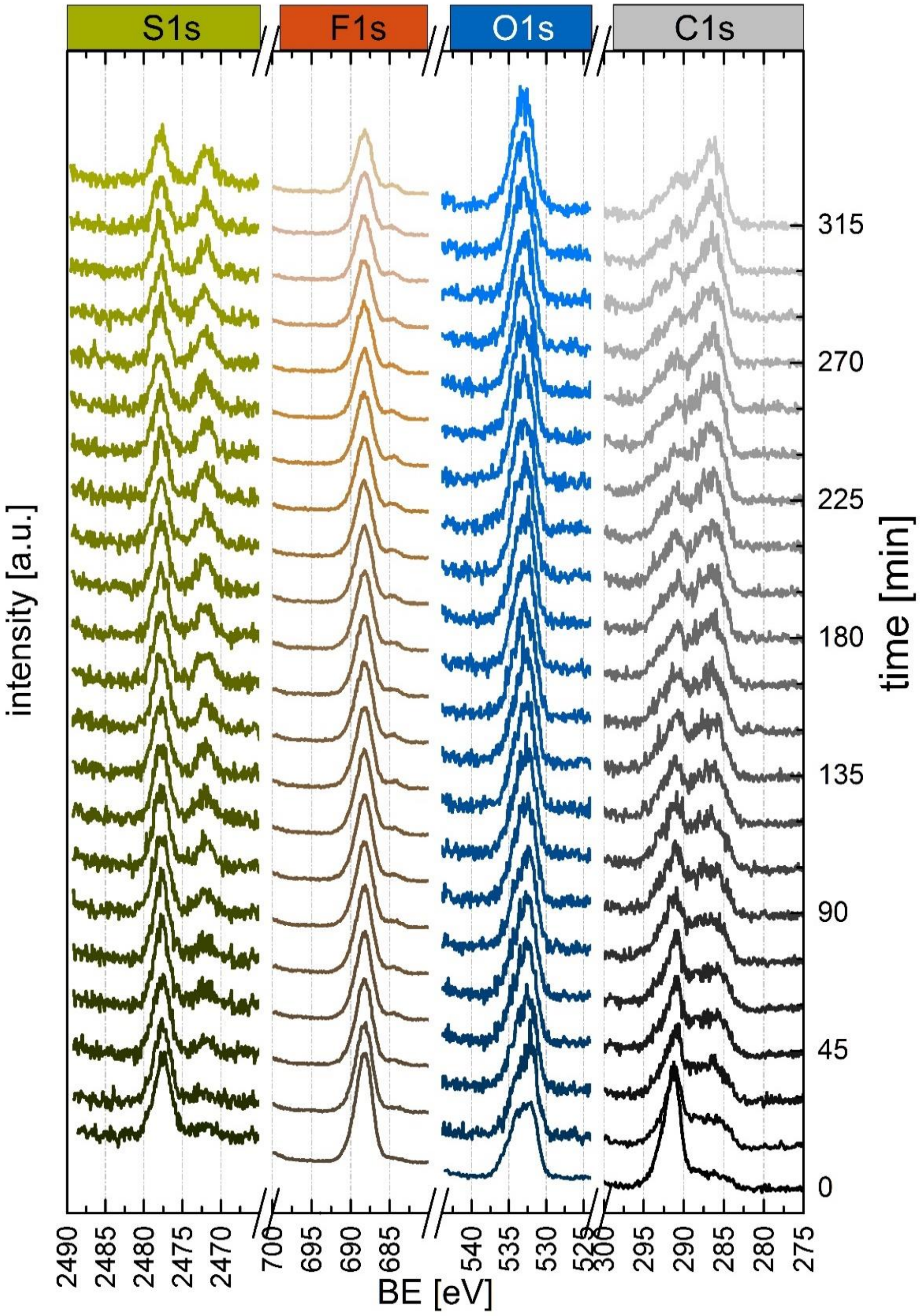

Figure S 9: Beam-induced damage of Li^+-exchanged Nafion™ HP membrane over time at an excitation energy of 5 keV. The change in the S 1s (green shades, outer left), F 1s (orange shades, inner left), O 1s (blue shades, inner right) and C 1s (grey shades, outer right) are shown. Energy calibration was executed based on the F 1s of the $–CF_2$ group of the polymer backbone.

As all changes occur gradually over time, a detailed discussion is based on the direct comparison of the pristine spectra and the spectra after more than 5 h of irradiation, which is shown in **Figure S 10**.

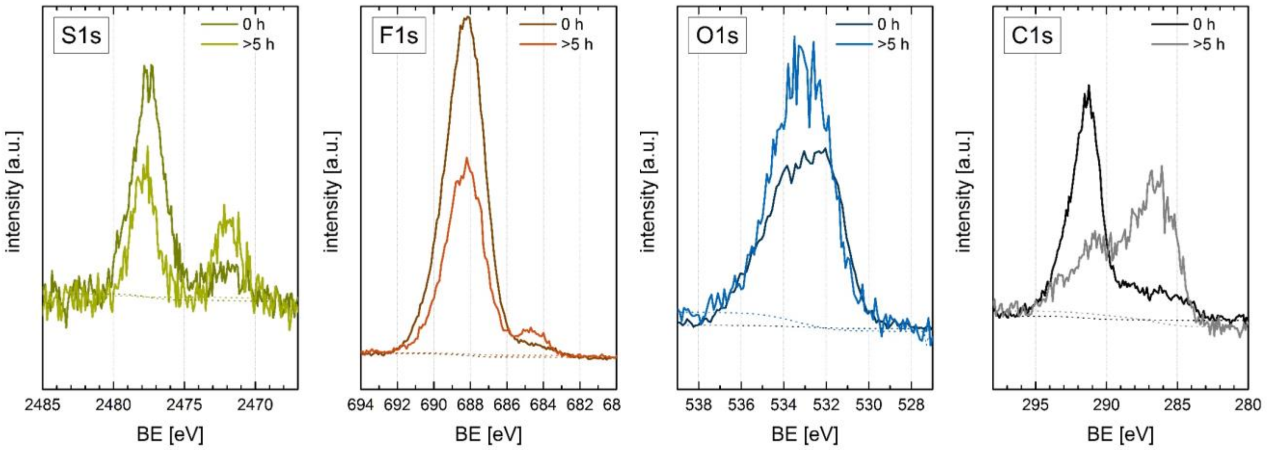

Figure S 10: Change of elemental spectra of Li^+-exchanged membrane over 5 h of irradiation in its dry state.

The chemical nature of the sulfur present as $–SO_3^-$ in the pristine MEA (left outer panel, dark green in **Figure S 10**) is partially changing upon prolonged irradiation. Roughly 55% of intensity is lost for the $–SO_3^-$ signal around 2477 eV in the S 1s spectra. At the same time, the low energy shoulder in the pristine material around 2471.5 eV rises continuously upon irradiation and at the end accounts for 40% of the sulfur signal in comparison to less than 10% in the pristine material. The overall amount of sulfur signal decreases by 33% throughout irradiation. For common lab-based XPS systems the S 2p orbital is probed, which is however too low in intensity at an X-ray energy of 5 keV as in this case. Based on the reference data provided by Yu et al.[14] employing a hard X-ray source, the peak in the S 1s region arising around 2471.5 eV corresponds most likely to a sulfide, thioether or adsorbed elemental sulfur. The latter would explain the overall loss in sulfur intensity as partial evaporation of sulfur might occur. Though the exact mechanism of this

chemical change in the $–SO_3$ group of PFSA upon prolonged irradiation can not be developed based on the limited data available, two reasonings can be stated. i) The ionic conductivity being provided by the $–SO_3$ group of the polymer is probably largely reduced at the irradiated spot (which will, however, not be reflected in the overall electrochemistry of the cell, as the probed area is very small in the *operando* APXPS measurement), which has already been observed for thin-film studies by Paul et al.[15] And ii) the removal of oxygen from the $–SO_3^-$ group upon irradiation will most likely be accompanied by oxidation of other parts of the polymer chain.

In contrast to the other spectra, the F 1s features remain rather constant upon irradiation (inner left panel in **Figure S 10**). The main peak at 688.3 eV corresponds to the dominant $–CF_2$ groups in the backbone and at the base of the side-chains in the Li^+-exchanged Nafion^{TM} membrane. It decreases by 45% in intensity whereas the small shoulder at lower BE (roughly 684.6 eV) corresponding to –CF– groups slightly increases. The loss in intensity along with the emergence of the low BE feature can be reasoned by the common "hydrolysis" reaction of Nafion^{TM} where HF is eliminated.[16] This reaction is triggered by the presence of radicals formed electrochemically in operating fuel cells and electrolyzers, but might as well be caused by excitation due to the X-ray beam, probably via the oxygen removal from the sulfonic acid group as discussed before.

The only element increasing overall in overall intensity is the O 1s. Due to the broad feature and significant noise observed as shown in the inner right panel of **Figure S 10**, a detailed analysis of the different components forming this peak is not possible. It seems however that parts of the low energy components have lost significantly in intensity. As the oxygen from the $–SO_3$ group is dominating this region, this observation goes along with the change in intensity of the main peak in the S 1s spectra.

The largest change upon irradiation is observed in the C 1s spectra (outer right panel in **Figure S 10**). While the pristine membrane spectrum (black line) is dominated by the $–CF_2$ feature at high BE and only a flat shoulder is observed at the low BE region, the membrane after irradiation (gray line) is dominated by low BE features. The high BE features ($–CF_x$) decrease by roughly 50% in intensity, which is in good agreement with the overall lower intensity of the F 1s signal upon irradiation. The peak observed at lower BE (centered around 286-287 eV) is very broad and most likely consists of a multitude of components that could include elemental carbon, thioether, –CH groups, and several carbon-oxygen compounds. The latter are most likely the dominating species, as the overall oxygen signal increased throughout irradiation. A smiliar chemical change in the C1 s of Nafion™ has been observed by Schulze et al. upon ion etching and X-ray exposure.[17]

Though the exact mechanism for beam-induced damage of Nafion™ is beyond the scope of this study, this reference measurement shows that the polymer is indeed undergoing large changes within the beam, most likely forming a film on the surface that is rich in carbon and oxygen. This could lead to the loss in sensitivity toward the active material that is covered by the polymer in the *operando* APXPS measurement (see **Figure S 8**). A polymer-based electrolyte that is more robust toward beam-induced damage would allow longer measurements and possibly higher resolved transition metal 2p spectra acquisition.

Besides beam-induced damage, anodic decomposition of the polymer electrolyte is a parasitic reaction that occurs during the *operando* measurement, especially as we are cycling to very high potentials of >5.2 V vs Li^+/Li. The anodic decomposition products could limit the possibility of tracking oxygen depletion from the oxide active material if they occurred in a similar energy range

in the O 1s XPS spectra. As our system contains no conductive salt, the decomposition products are limited to the products of anodic decomposition of PC (that inevitably takes place minding the strong CO_2 evolution observed during the OEMS measurements) and possibly products of PFSA decomposition at high potentials. As shown later in this section, the F 1s and S 1s and C 1s spectra of the *operando* measurement do not change significantly upon charging to high potentials, still a model experiment was executed in order to exclude interference with the O 1s analysis of the oxide region. For this, a MEA with a pure C65 working electrode was fabricated (i.e., without any layered oxide active material). The same experimental procedure as for the NCM containing electrode was used, whereas the solid ratio in the electrode ink was 10/4 for C65/ LITHion^{TM} in order to keep the polymer film thickness on the carbon constant. After 12 h of soaking in the *operando* XPS cell (without the milled conically slit in the top plate), this MEA was charged in the GB with 1 mV/s to a cell potential of 1.7 V (i.e., 5.2 V vs Li^+/Li) and held there for 2 h. Afterwards the MEA was inertly transferred into a glass oven and dried at 80°C under vacuum over night. XPS spectra were recorded using a lab based XPS (Kratos Axis Supra spectrometer using monochromatic Al Kα radiation at an energy of 1486.6 eV) at $3 \cdot 10^{-8}$ Torr in comparison to the pristine C65 electrode. The comparison of the O 1s spectra in **Figure S 11** should be a good indicator as to whether electrolyte decomposition products could interfere with the oxide region in the *operando* XPS data set.

Comparing the O 1s XPS characteristics before (black line) and after (orange line) anodic decomposition of the polymer electrolyte, it becomes evident, that the oxide region is not majorly affected by possible decomposition products during the *operando* XPS measurement. A broadening of the electrolyte-assigned peaks mainly due to an increase in the mid energy feature region is observed that could explain the *operando* data set at low SOC to show a slight change in

spectral shape before emergence of the oxygen-depleted phase. In this region, C-O containing decomposition products (most likely stemming from anodic oxidation of PC) are present.[18] It has to be noted that the effect of these decomposition products on the overall O 1s intensity and shape is aggravated by the model electrode approach due to the significantly higher surface area of the C65 that leads to higher amounts of PC getting decomposed and due to the measurement conditions in high vacuum resulting in loss in "unaltered" O 1s components stemming from liquid PC. Changes in the S 2p and F 1s spectra have not been observed in this model experiment in accordance to the next paragraph discussing chemical changes in the electrolyte components during the *operando* XPS measurement. This model experiment shows that electrolyte decomposition products do not interfere with the *operando* analysis of the oxide region and that the additional feature emerging roughly 1 eV higher in BE than the layered oxide O 1s signal stems indeed from the active material.

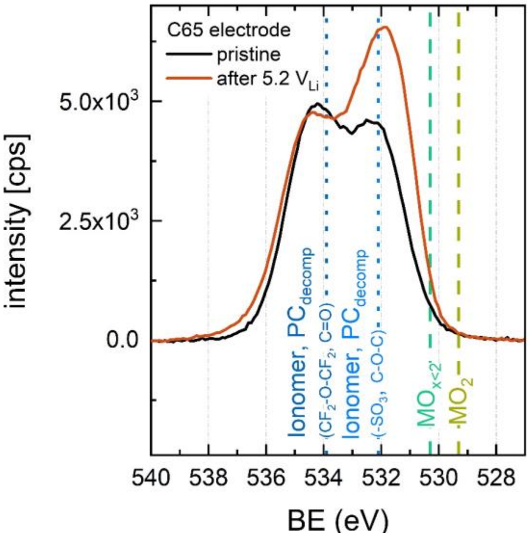

Figure S 11: Change of background corrected O 1s signals of a C65 model electrode upon anodic polarization. Measured in a high vacuum XPS system (i.e., without contribution of liquid PC).

In order to check for anodic oxidation and severity of beam-induced damage of the polymer electrolyte during the *operando* measurement, we compare the evolution of the C 1s, F 1s and S 1s in **Figure S 12**.

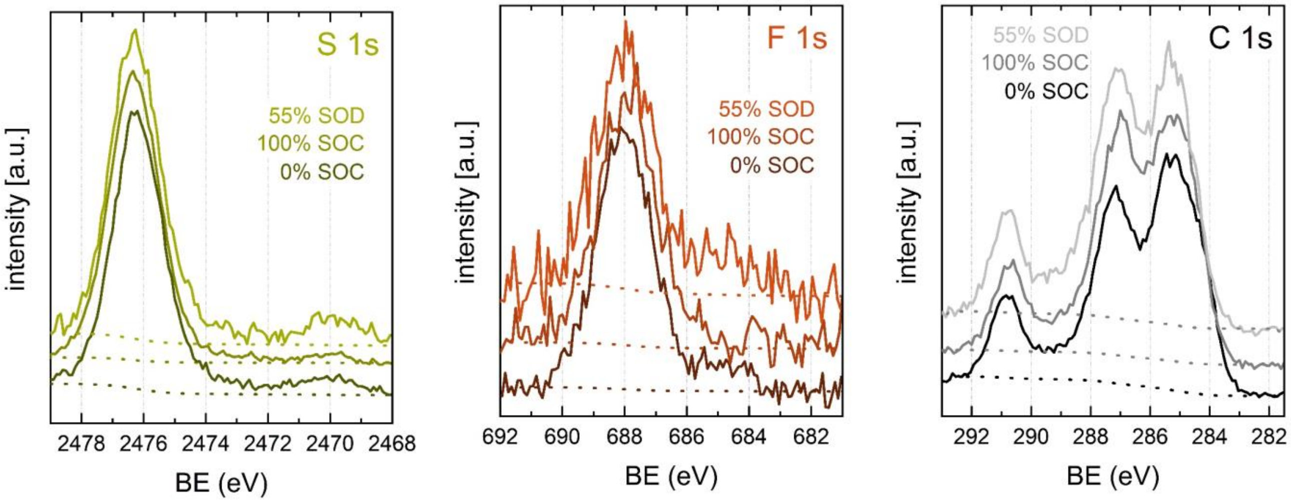

Figure S 12: Evolution of the electrolyte component-bearing S 1s, F 1s and C 1s spectra of the *operando* XPS measurement.

From the C 1s, F 1s and S 1s spectra obtained during the *operando* XPS measurement, no evidence for the anodic decomposition of the PFSA can be inferred. Slight changes observed in the low BE region for the F 1s and S 1s indicate minor beam-induced damage (see also **Figure S 10**) but as the shoulders rising around 685 eV in the F 1s and around 2470 eV in the S 1s are not potential dependent (i.e., the spectrum recorded at 100% SOC shows the least pronounced feature), this difference is caused by slight variations in the time-span of irradiation rather than it being indicative of an anodic oxidation process.

S7. OEMS Measurements and Additional Experimental Methods

In order to mimic the *operando* XPS cell, the bottom of the OEMS cell was equipped with a stack of stainless steel plates (15 mm_{dia}) to center the GF rings used as a PC reservoir while also enabling electrical contact to the LFP counter electrode. On top of the MEA, a stainless steel mesh (SS316, aperture 1 mm, wire diameter 0.22 mm, Spörl, Germany) is added as current collector for the NCM111 working electrode, thereby still allowing direct probing of the gas formed during cycling.

The OEMS cells were assembled in an argon filled GB (MBraun, <0.1 ppm H_2O, <0.1 ppm O_2). The closed cell was removed from the GB and transferred to the OEMS system. The gases evolved during cycling of the MEA are guided to the mass spectrometer via a flow restricting capillary. Prior to cycling, there was a 12 h period at open circuit voltage (OCV) to allow for complete soaking of the polymer with the PC solvent, which was also used to allow extrapolation of the background of the mass spectrometers m/z signals.

As our OEMS is a 2-electrode set-up, the cut-off potentials were set based on the cell potential. The symmetric overpotential of the LFP counter electrode was determined experimentally by reference measurements (see section S1 of the SI) to be 3.5±0.1 V and 3.5±0.3 V when cycling the MEA at C/30 and C/10, respectively. After cycling, the cell was flushed with a calibration gas of 2000 ppm of O_2 and 2000 ppm of CO_2 in argon to quantify the evolved amounts of those gases at m/z=32 and m/z=48, respectively.

Considering the OEMS gas evolution characteristics of the MEA cycled at C/30 in **Figure 2 a** of the main text, the evolution of CO_2 sets in at roughly 4.6 V vs Li^+/Li (bottom panel, blue line), which is a commonly reported value for the anodic oxidation of organic carbonate based electrolytes and/or their impurities.[19-22] The O_2 onset (green line) is observed slightly later when

the NCM electrode is delithiated to roughly 75% SOC, which agrees well with standard gassing measurements and is known to arise from the crystal lattice destabilization at high degrees of delithiation leading to O_2 evolution from the near-surface region of NCM particles.[19, 23-24] While the CO_2 onset for the intermittent cycling procedure shown in **Figure 2 b** also arises at roughly 4.6 V vs Li^+/Li (blue line, bottom panel), the O_2 onset is already observed at the beginning of the constant current charge going from 60% to 80% SOC (green line). Although the major portion of O_2 is evolved in the last step, going from 80% SOC to 100% SOC, this earlier gassing onset shows that in the battery MEA set-up, a C-rate of C/10 already leads to local inhomogeneity of the SOC of the NCM111 electrode. This must stem from *inter*particular resistances, as *intra*particular limitations would be independent of whether the MEA set-up or a standard liquid cell is used. While this resistance is inherently linked to the MEA set-up used in this study, the overall electrochemical inhomogeneity is small and cycling the *operando* APXPS cell at C/10 is therefore thought to provide representative findings for studying the NCM electrode material.

The OEMS gas evolution analysis shows only that an inhomogeneous SOC distribution is present when cycling the NCM111 battery MEA at C/10; it is not possible to differentiate whether the electrical or the ionic conductivity causes this behavior. For a discussion on the limiting charge carrier resistance, please see discussion in section S1 and S2.

S8. Comparison of *Ex-situ* and *Operando* O 1s Spectra

In order to validate the results obtained during the *operando* APXPS measurement, a comparison is drawn to a standard *ex-situ* sample measured at a lab-based XPS instrument and in the *operando* APXPS set-up. Several parameters could have influenced the *operando* data: i) a high flux X-ray source at 5 keV might have caused beam-damage already within the short time frame of data collection at each SOC spot. ii) data was collected in the open area of the spectro-electrochemical cell that, in combination with the reduced pressure in the analysis chamber, might lead to an electrochemically inactive area to be studied. iii) the combination of the electrolyte experiencing high anodic polarization and X-ray attenuation concomitantly could lead to a parasitic product formed by photolysis and concurrent anodic oxidation or vice versa.

We therefore first compare the O 1s spectra obtained from an *ex-situ* sample at a lab-based XPS (Kratos Axis Supra spectrometer using monochromatic Al Kα radiation at an energy of 1486.6 eV) at $3 \cdot 10^{-8}$ Torr with the *operando* data for a pristine (0% SOC) and fully charge (100% SOC) NCM111 MEA. The *ex-situ* reference was either only soaked in PC for 12 h in an argon-filled GB (MBraun, <0.3 ppm H_2O, <0.1 ppm O_2) or cycled in the GB using a cell with a closed lid in order to make sure that 100% of the working electrode area is electrically connected. The electrochemical response of the *ex-situ* reference is shown in **Figure S 2**. All *ex-situ* references were disassembled in the GB, inertly transferred into a glass oven and dried at 120°C under vacuum for 5 days before inert transfer into the interlock of the lab-based XPS.

In **Figure S 13** several differences between O 1s spectra of the *ex-situ* reference measured at high vacuum (top 2 spectra) and the *operando* data obtained (bottom left and central spectra) can be observed. The overall lower relative intensity of the electrolyte based feature in the *ex-situ* data

can be assigned to the measurement at high-vacuum and the absence of the PC solvent. For the 100% SOC *ex-situ* samples the increase of the low BE electrolyte component can be correlated to the model experiment using C65 where an increase in intensity around 532 eV was observed, which is linked to the formation of electrolyte decomposition products. A difference in the relative intensity of the oxide region and the electrolyte region between the *ex-situ* data and *operando* data is caused by the sample preparation time, lack of organic solvent in the high vacuum XPS system and lower attenuation length caused by the different X-ray source.

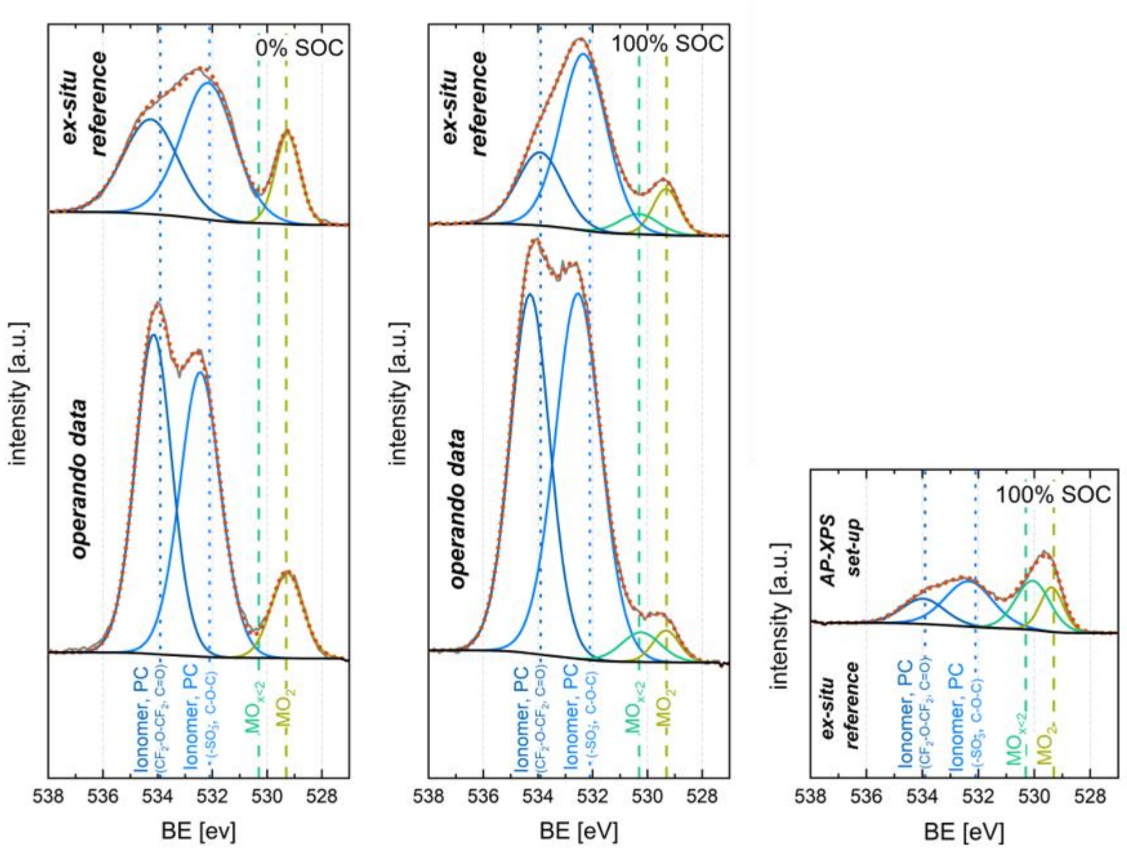

Figure S 13: O 1s spectra comparison of MEA cycled *ex-situ* and measured at a standard lab-based XPS (top) with data obtained in the APXPS set-up at the ALS, BL 9.3.1 (bottom) at 0% SOC (left) and 100% SOC (center and right). The spectra in the bottom center is spectrum 6 of the *operando* data set (see section S5). The spectra at the bottom right corresponds to a NCM111 MEA charged to 100% SOC as *ex-situ* sample but measured at the APXPS endstation using 5 keV excitation energy. Spectra are normalized to the intensity of the oxide components (MO_2+$MO_{x<2}$).

Nevertheless, for both systems when comparing the pristine and charged state, the emergence of an additional component in the O 1s spectra slightly higher in BE than the layered oxide material can be observed. This direct comparison shows that the characteristics of our *operando* data are comparable to the standard *ex-situ* method and that the spectra collected can be directly linked to the SOC dependent oxygen environment at the active material particle surface. This setup will therefore enable us to do more complex analysis of time, potential, and depth dependent processes.

Though the *ex-situ* references experienced extensive rest time before analysis either in the high-vacuum lab-based XPS instrument or at the APXPS endstation at ALS BL 9.3.1, we want to give an estimate on the *M*:O ratio in order to compare it to the numbers obtained during the *operando* APXPS measurement.

First we estimate the probing depth for the lab-based XPS instrument. Besides the change in excitation energy and therewith change in the inelastic mean free path (IMFP) of photoelectrons with a kinetic energy of roughly 956 eV (i.e., stemming from the O 1s orbital), the absence of the PC solvent for the *ex-situ* data is taken into account.

For the dry polymer covering the active material at 0% SOC (**Figure S13,** top left spectra) we can use Equation 8 to calculate the overlayer thickness. Considering the change in IMFP of electrons in the dry electrolyte polymer and the active material in the lab-based XPS instrument of $\lambda_{ely}^{dry} = 2.67\ nm$ and $\lambda_{MO_2} = 2.14\ nm$ (obtained using the *QUASES-IMFP calculation by TPP2m formula* software), the overlayer thickness of the dry electrolyte can be calculated to $d_{ely}^{dry} = 4.1\ nm$ (considering the necessary electrolyte correction as given in Equation 9). Minding the limitations of the overlayer calculation to be derived for flat surfaces, this value is seen as a good agreement to the calculated value of 5.2 nm of dry polymer film thickness obtained with Equation 3.

Now we want to estimate the thickness of the oxygen-depleted layer of the *ex-situ* sample charged to 100% SOC measured at the lab-based XPS system (**Figure S13,** top central spectra). First we calculate the *M*:O ratio after oxygen depletion taking into account the lower resolution and the different relative sensitivity factor of the lab-based instrument for the Ni $2p_{3/2}$ spectra ($rsf^{O\,1s}_{Ni\,2p_{3/2}} \approx 4.6$) end up at M:O $\approx 0.63 \pm 0.21$. This uncertainty for the *M*:O ratio is significantly higher for the lab-based XPS instrument compared to the APXPS set-up using an excitation energy of 5 keV.

A comparison of the values obtained in the *operando* APXPS measurement at the end of charge (spectra 6), at the end of discharge (spectra 9) and the *ex-situ* measurements from a sample charged to 100% SOC before spectral analysis is given in **Table S5**.

**O 1s spectrum**		**Areal ratio** $\frac{I_{MO_{x<2}}}{I_{MO_2}}$	***M*:O ratio** *measured*	**M_3O_4 spinel formation**		**MO rock-salt formation**	
				$d_{MO_{x<2}}$ [nm]	*M*:O ratio *expected*	$d_{MO_{x<2}}$ [nm]	*M*:O ratio *expected*
6 – 100% SOC		1.41	0.62 ±0.08	6.2	0.64	6.5	0.79
9 – 55% SOD		1.90	0.65 ±0.08	7.5	0.66	7.7	0.83
Ex-situ – 100% SOC	Lab-based XPS	0.65	0.63 ±0.21	1.1	0.60	1.1	0.70
	ALS 9.3.1	1.63	0.62 ±0.05	6.8	0.65	7.1	0.81

Table S 5: Comparison of oxygen-depleted surface layer formation of *operando* and *ex-situ* data.

While the thickness of the oxygen-depleted surface layer of the NCM111 active material is on the same order of magnitude for the *operando* and *ex-situ* data sets, the values still differ significantly.

One possible explanation would be that the battery MEA cycled in the beam undergoes surface oxygen-depletion in a different manner or to a different extent compared to cycling at ambient-

pressure and without possible beam-induced parasitic reactions. In order to exclude such influences caused by the experimental set-up, a second *ex-situ* reference was prepared as described before (including drying at 120°C) and measured at the beam-line with the same parameters as for the *operando* measurement. Upon removal of the PC solvent the contribution of the metal oxide region to the overall O 1s intensity is improved, which can be seen in **Figure S13**, bottom right spectra. Looking at the values obtained for the relative intensity ratio of the two oxide phases in the O 1s $\frac{I_{MO_{x<2}}}{I_{MO_2}}$ and the *M*:O ratio we can again calculate the overlayer thickness of the oxygen-depleted phase as given in **Table S5**. Comparing this *ex-situ* data to the *operando* data set we conclude that the difference in estimated overlayer thickness is likely caused by the different measurement set-ups in combination with the linear attenuation model. We therefore see our findings of the *operando* APXPS experiment meaningful and exclude significant alteration of the surface changes happening upon cycling due to the special measurement set-up.

At this point we can not give a physically based reason for the different layer thicknesses obtained on the lab-based XPS and the APXPS set-up, but a possible hypothesis is the limitation of the linear attenuation model used to estimate the film thickness upon oxygen-depletion, possibly in combination with the sample preparation for the *ex-situ* sample.

ABBREVIATIONS

ALS, Advances Light Source; BE, binding energy; EIS, electrochemical impedance spectroscopy; GB, glovebox; IMFP, inelastic mean free path; MEA, membrane electrode assembly; OEMS, online electrochemical mass spectrometry; PC, propylene carbonate; SOC, state-of-charge; SOD, state-of-discharge; XPS, X-ray photoelectron spectroscopy.

REFERENCES

1. Doyle, M.; Lewittes, M. E.; Roelofs, M. G.; Perusich, S. A., Ionic Conductivity of Nonaqueous Solvent-Swollen Ionomer Membranes Based on Fluorosulfonate, Fluorocarboxylate, and Sulfonate Fixed Ion Groups. *The Journal of Physical Chemistry B* **2001,** *105*, 9387-9394.

2. Nasirzadeh, K.; Neueder, R.; Kunz, W., Vapor Pressures of Propylene Carbonate and N,N-Dimethylacetamide. *Journal of Chemical & Engineering Data* **2005,** *50*, 26-28.

3. Pokorný, V.; Štejfa, V.; Fulem, M.; Červinka, C.; Růžička, K., Vapor Pressures and Thermophysical Properties of Ethylene Carbonate, Propylene Carbonate, γ-Valerolactone, and γ-Butyrolactone. *Journal of Chemical & Engineering Data* **2017,** *62*, 4174-4186.

4. Etxebarria, A.; Yun, D. J.; Blum, M.; Ye, Y.; Sun, M.; Lee, K. J.; Su, H.; Munoz-Marquez, M. A.; Ross, P. N.; Crumlin, E. J., Revealing In Situ Li Metal Anode Surface Evolution upon Exposure to CO2 Using Ambient Pressure X-Ray Photoelectron Spectroscopy. *ACS Appl Mater Interfaces* **2020,** *12*, 26607-26613.

5. Sicklinger, J.; Metzger, M.; Beyer, H.; Pritzl, D.; Gasteiger, H. A., Ambient Storage Derived Surface Contamination of NCM811 and NCM111: Performance Implications and Mitigation Strategies. *J. Electrochem. Soc.* **2019,** *166*, A2322-A2335.

6. Friedrich, F.; Strehle, B.; Freiberg, A. T. S.; Kleiner, K.; Day, S. J.; Erk, C.; Piana, M.; Gasteiger, H. A., Editors' Choice—Capacity Fading Mechanisms of NCM-811 Cathodes in Lithium-Ion Batteries Studied by X-ray Diffraction and Other Diagnostics. *J. Electrochem. Soc.* **2019,** *166*, A3760-A3774.

7. Maibach, J.; Kallquist, I.; Andersson, M.; Urpelainen, S.; Edstrom, K.; Rensmo, H.; Siegbahn, H.; Hahlin, M., Probing a battery electrolyte drop with ambient pressure photoelectron spectroscopy. *Nat Commun* **2019,** *10*, 3080.

8. Friedman, A. K.; Shi, W.; Losovyj, Y.; Siedle, A. R.; Baker, L. A., Mapping Microscale Chemical Heterogeneity in Nafion Membranes with X-ray Photoelectron Spectroscopy. *J. Electrochem. Soc.* **2018,** *165*, H733-H741.

9. Ehlert, C.; Kröner, D.; Saalfrank, P., A combined quantum chemical/molecular dynamics study of X-ray photoelectron spectra of polyvinyl alcohol using oligomer models. *J. Electron. Spectrosc. Relat. Phenom.* **2015,** *199*, 38-45.

10. Awatani, T.; Midorikawa, H.; Kojima, N.; Ye, J.; Marcott, C., Morphology of water transport channels and hydrophobic clusters in Nafion from high spatial resolution AFM-IR spectroscopy and imaging. *Electrochem. Commun.* **2013,** *30*, 5-8.

11. Jung, R.; Morasch, R.; Karayaylali, P.; Phillips, K.; Maglia, F.; Stinner, C.; Shao-Horn, Y.; Gasteiger, H. A., Effect of Ambient Storage on the Degradation of Ni-Rich Positive Electrode Materials (NMC811) for Li-Ion Batteries. *J. Electrochem. Soc.* **2018,** *165*, A132-A141.

12. Ruess, R.; Schweidler, S.; Hemmelmann, H.; Conforto, G.; Bielefeld, A.; Weber, D. A.; Sann, J.; Elm, M. T.; Janek, J., Influence of NCM Particle Cracking on Kinetics of Lithium-Ion Batteries with Liquid or Solid Electrolyte. *J. Electrochem. Soc.* **2020,** *167*.

13. Oswald, S.; Pritzl, D.; Wetjen, M.; Gasteiger, H. A., Novel Method for Monitoring the Electrochemical Capacitance by In Situ Impedance Spectroscopy as Indicator for Particle Cracking of Nickel-Rich NCMs: Part I. Theory and Validation. *J. Electrochem. Soc.* **2020,** *167*.

14. Yu, L.; Takagi, Y.; Nakamura, T.; Sakata, T.; Uruga, T.; Tada, M.; Iwasawa, Y.; Masaoka, S.; Yokoyama, T., Operando Observation of Sulfur Species Poisoning Polymer Electrolyte Fuel Cell Studied by Near Ambient Pressure Hard X-ray Photoelectron Spectroscopy. *The Journal of Physical Chemistry C* **2018,** *123*, 603-611.

15. Paul, D. K.; Giorgi, J. B.; Karan, K., Chemical and Ionic Conductivity Degradation of Ultra-Thin Ionomer Film by X-ray Beam Exposure. *J. Electrochem. Soc.* **2013,** *160*, F464-F469.

16. Fruhwirt, P.; Kregar, A.; Torring, J. T.; Katrasnik, T.; Gescheidt, G., Holistic approach to chemical degradation of Nafion membranes in fuel cells: modelling and predictions. *Phys. Chem. Chem. Phys.* **2020,** *22*, 5647-5666.

17. Schulze, M.; Lorenz, M.; Wagner, N.; Gülzow, E., XPS analysis of the degradation of Nafion. *Fresenius' Journal of Analytical Chemistry* **1999,** *365*, 106-113.

18. Lebens-Higgins, Z. W.; Chung, H.; Zuba, M. J.; Rana, J.; Li, Y.; Faenza, N. V.; Pereira, N.; McCloskey, B. D.; Rodolakis, F.; Yang, W.; Whittingham, M. S.; Amatucci, G. G.; Meng, Y. S.; Lee, T. L.; Piper, L. F. J., How Bulk Sensitive is Hard X-ray Photoelectron Spectroscopy: Accounting for the Cathode-Electrolyte Interface when Addressing Oxygen Redox. *J Phys Chem Lett* **2020,** *11*, 2106-2112.

19. Strehle, B.; Kleiner, K.; Jung, R.; Chesneau, F.; Mendez, M.; Gasteiger, H. A.; Piana, M., The Role of Oxygen Release from Li- and Mn-Rich Layered Oxides during the First Cycles Investigated by On-Line Electrochemical Mass Spectrometry. *J. Electrochem. Soc.* **2017,** *164*, A400-A406.

20. Solchenbach, S.; Metzger, M.; Egawa, M.; Beyer, H.; Gasteiger, H. A., Quantification of PF_5 and POF_3 from Side Reactions of $LiPF_6$ in Li-Ion Batteries. *J. Electrochem. Soc.* **2018,** *165*, A3022-A3028.

21. Freiberg, A. T. S.; Sicklinger, J.; Solchenbach, S.; Gasteiger, H. A., Li_2CO_3 decomposition in Li-ion batteries induced by the electrochemical oxidation of the electrolyte and of electrolyte impurities. *Electrochim. Acta* **2020,** *346*, 136271.

22. Hatsukade, T.; Schiele, A.; Hartmann, P.; Brezesinski, T.; Janek, J., Origin of Carbon Dioxide Evolved during Cycling of Nickel-Rich Layered NCM Cathodes. *ACS Appl Mater Interfaces* **2018,** *10*, 38892-38899.

23. Jung, R.; Metzger, M.; Maglia, F.; Stinner, C.; Gasteiger, H. A., Oxygen Release and Its Effect on the Cycling Stability of $LiNi_xMn_yCo_zO_2$ (NMC) Cathode Materials for Li-Ion Batteries. *J. Electrochem. Soc.* **2017,** *164*, A1361-A1377.

24. Wandt, J.; Freiberg, A. T. S.; Ogrodnik, A.; Gasteiger, H. A., Singlet oxygen evolution from layered transition metal oxide cathode materials and its implications for lithium-ion batteries. *Mater. Today* **2018,** *21*, 825-833.
